# Triterpenes from *Pholiota populnea* as Cytotoxic Agents and Chemosensitizers to Overcome Multidrug Resistance of Cancer Cells

**DOI:** 10.1021/acs.jnatprod.1c01024

**Published:** 2022-03-16

**Authors:** Morteza Yazdani, Zoltán Béni, Miklós Dékány, Nikoletta Szemerédi, Gabriella Spengler, Judit Hohmann, Attila Ványolós

**Affiliations:** † Department of Pharmacognosy, Interdisciplinary Excellence Centre, University of Szeged, 6720 Szeged, Hungary; ‡ Spectroscopic Research Department, Gedeon Richter Plc., Gyömrői út 19-21, H-1103 Budapest, Hungary; § Department of Medical Microbiology, Albert Szent-Györgyi Health Center and Faculty of Medicine, University of Szeged, Semmelweis utca 6, H-6725 Szeged, Hungary; ∧ Interdisciplinary Centre for Natural Products, University of Szeged, Eötvös u. 6, H-6720 Szeged, Hungary; ∥ Department of Pharmacognosy, Semmelweis University, Üllői u. 26, H-1085 Budapest, Hungary

## Abstract

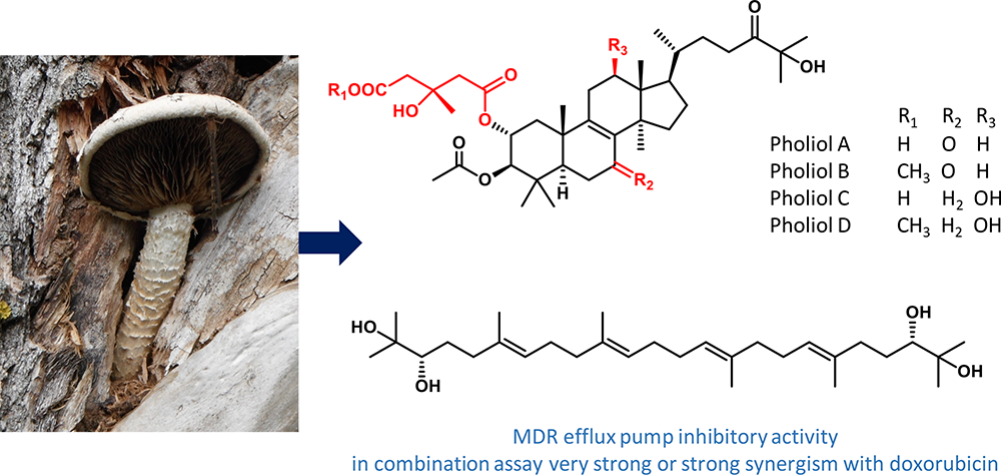

The detailed mycochemical analysis of the *n*-hexane extract of *Pholiota populnea* led to the isolation of four new lanostane diesters, named pholiols A–D (**1**–**4**), together with an acyclic triterpene, (3*S*,6*E*,10*E*,14*E*,18*E*,22*S*)-2,3,22,23-tetrahydroxy-2,6,10,15,19,23-hexamethyl-6,10,14,18-tetracosatetraene (**5**), ergosterol (**6**), and 3β-hydroxyergosta-7,22-diene (**7**). The isolation was carried out by multistep flash chromatography, and the structures were elucidated using extensive spectroscopic analyses, including 1D and 2D NMR and MS measurements. The isolated metabolites (**1**–**6**) were investigated for cytotoxic activity against Colo205 and Colo320 colon adenocarcinoma and nontumoral MRC-5 cell lines. Among the tested compounds, ergosterol (**6**) showed substantial cytotoxic activity against all cell lines with IC_50_ values of 4.9 μM (Colo 205), 6.5 μM (Colo 320), and 0.50 μM (MRC) with no tumor cell selectivity. A P-glycoprotein efflux pump modulatory test on resistant Colo320 cells revealed that pholiols A (**1**) and B (**2**) and linear triterpene polyol **5** have the capacity to inhibit the efflux-pump overexpressed in the cells. Moreover, the drug interactions of triterpenes with doxorubicin were studied by the checkerboard method on Colo 320 cells. Pholiols B (**2**) and D (**4**) interacted in synergistic and acyclic triterpene **5** in a very strong synergistic manner; the combination index (CI) values at 50% of the growth inhibition dose (ED_50_) were found to be 0.348, 0.660, and 0.082, respectively. Our results indicate that *P. populnea* is a promising source for finding new triterpenes with significant chemosensitizing activity on cancer cells.

Cancer is among the leading causes of morbidity and mortality worldwide. According to Global Cancer Statistics, an estimated 19.3 million new cancer cases, among them 1.148 million new colon cancer instances (representing 6.0% of all cases), were registered worldwide in 2020.^1^ A major problem in clinical cancer therapy is the multidrug resistance (MDR) toward cytotoxic drugs. MDR is associated with cellular pharmacokinetic alterations, such as decreased drug accumulation, increased efflux and detoxification capacity, and subcellular redistribution. One of the most widespread mechanisms of resistance involves the efflux of drug molecules out of the cells. In particular, ATP-binding cassette (ABC) transporters have received great interest; one such transporter that has been investigated in detail is P-glycoprotein (P-gp).^2^ Triterpenoids were reported to be able to reverse cell resistance to chemotherapy due to P-gp inhibition. Ginsenoside Rh2, isolated from red ginseng, exerts such activity in doxorubicin-resistant human breast cancer MCF-7 cells.^3^ Oleanolic acid was reported to be an inhibitor of certain efflux transporters, including P-gp35, and thus was found to increase the intracellular concentration of paclitaxel.^4^ Maslinic acid, a natural triterpene from *Olea europaea* L., has attracted increasing interest in recent years because of its promising anticancer activity and dose-dependent enhancing potency of docetaxel (DOC) sensitivity and cellular drug accumulation in MDA-MB-231/DOC cells in a combination treatment.^5^ The tetracyclic triterpene alcohol euphol was reported to have cytotoxicity (IC_50_ < 10 μM) against 27 cell lines when screened against a panel of 71 human cancer cells from 15 tumor types. This compound also exhibited antitumoral and antiangiogenic activity *in vivo*, with synergistic temozolomide interactions in most cell lines.^6^ The pentacyclic terpenoid boswellic acid was studied in combination with doxorubicin for the antitumor effects against solid tumors of Ehrlich’s ascites carcinoma grown in mice. The results showed that boswellic acid synergized the antitumor activity of doxorubicin.^7^ The fungal metabolite ergosta-7,22-diene-3-one was found to be effective against sensitive (Colo 205) and resistant (Colo 320) human colon adenocarcinoma cells with IC_50_ values 11.6 ± 1.7 μM and 8.4 ± 1.1 μM, respectively, and in a combination assay synergism was detected between ergosta-7,22-diene-3-one and doxorubicin with CI index 0.521 ± 0.15 at the 50% growth inhibition dose (ED_50_).^8^

*Pholiota populnea* (Pers.) Kuyper & Tjall.-Beuk. (syn. *Pholiota destruens* (Brond.) Quel., *Hemipholiota populnea*) is member of the Strophariaceae family, distributed worldwide wherever cottonwood occurs. This mushroom species is usually saprophytic, but sometimes parasitic, and grows on broad-leaved woods, mainly on various poplars, but also on willow and birch, playing an important role in decomposing the deadwood of cottonwoods. The chemistry and pharmacology of *P. populnea* were poorly studied previously, with only pholiotic acid, 3,5-dichloro-4-methoxybenzaldehyde, and 3,5-dichloro-4-methoxybenzyl alcohol, with weak antifungal and cytostatic activities, identified.^9^ In a recent paper the mineral element composition of this species was reported.^10^

In the current study, the detailed mycochemical analysis of a methanolic extract of *H. populnea*, isolation, structure determination, and pharmacological evaluation of its chemical compounds are highlighted. The present paper reports four new lanostane diesters (**1**–**4**), one acyclic triterpene tetraol (**5**), and the known compounds ergosterol (**6**) and 3β-hydroxyergosta-7,22-diene (**7**). The isolated compounds **1**–**6** were investigated for cytotoxic activity against human colon adenocarcinoma cells (Colo205, Colo320) and the MRC-5 cell line. The combination with doxorubicin and efflux pump inhibitory activity of the compounds on drug-resistant Colo 320 cells were also assayed. In addition, antimicrobial activity against different bacterial strains was evaluated by the microdilution method.

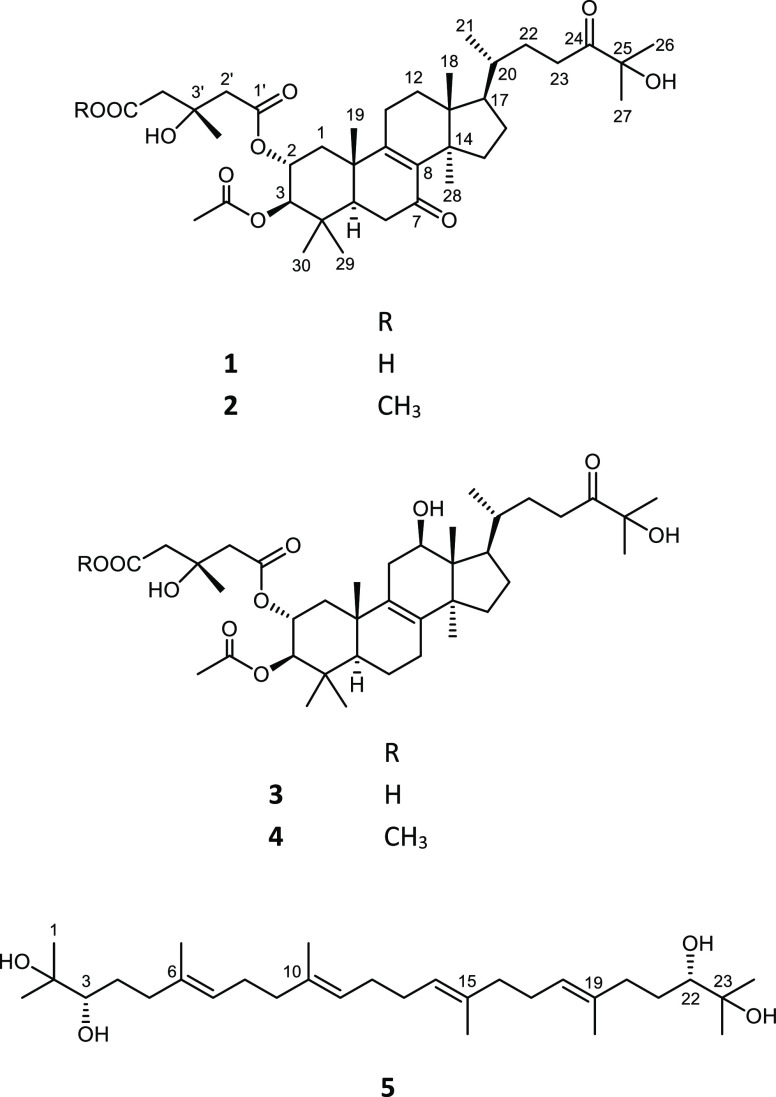



## Results and Discussion

Four lanostane triterpenes (**1**–**4**) and an acyclic triterpene tetraol (**5**) were isolated from the *n*-hexane-soluble phase of the MeOH extract prepared from the mushroom *P. populnea* by a combination of multiple flash chromatography steps. The structure elucidation was carried out by extensive spectroscopic analysis, including 1D and 2D NMR (^1^H–^1^H COSY, HSQC, HMBC, and ROESY) and HRESIMS experiments.

Based on the HRESIMS data, the molecular formulas of compounds **1** and **2** were C_38_H_58_O_10_ and C_39_H_60_O_10_, respectively, differing only in a methylene group. The ^1^H and ^13^C NMR spectra of **1** and **2** (Tables 1 and 2) were similar except for a singlet signal with three-proton intensity at δ_H_ 3.71 appearing in the ^1^H NMR spectrum and the extra resonance at δ_C_ 51.7 in the ^13^C NMR spectrum of **2**. These findings suggested that **2** was the *O*-methyl derivative of **1**. In accordance with the elemental compositions, the ^13^C NMR spectra presented 38 and 39 resonances in compounds **1** and **2**, respectively. Based on the HSQC spectra, besides the additional methoxy group in **2**, 10 methyl, 10 methylene, and 5 methine groups, and 13 nonprotonated carbons were commonly present in the compounds. Considering the elemental compositions and the chemical shift values of the nonprotonated carbons, two carbonyl groups (δ_C_ 214.9/214.8 and δ_C_ 197.9/197.9), three carboxylate moieties (δ_C_ 173.7/172.1, 170.8/171.2, and 171.4/170.6), an unsaturation (δ_C_ 139.2/139.1 and δ_C_ 163.1/163.0), and two sp^3^ carbons attached to oxygens (δ_C_ 76.2/76.2 and 69.6/69.5) were present in **1** and **2**. The remaining four signals (δ_C_ 39.3/39.2, 40.5/40.5, 44.9/44.9, and 47.8/47.8 for **1**/**2**) could be ascribed to sp^3^ nonprotonated carbons. Based on the characteristic HMBC correlations of the angular methyl singlets [H-18 with C-12, C-13, C-14, and C-17; H-19 with C-1, C-5, C-9, and C-10; H-21 with C-17, C-20, and C-22; H-29 and H-30 with C-3, C-4, and C-5], it was concluded that **1** and **2** are based on a lanostane skeleton. The HMBC correlations of the *O*-methine doublets at δ_H_ 4.85/4.83 with C-1, C-4, C-29, and C-30 confirmed their assignment to H-3 in **1**/**2**, while HMBC cross-peaks of the *O*-methine groups at δ_C_ 70.3/69.9 (C-2) with protons at δ_H_ 4.85/4.83 (H-3) suggested that the lanosterol skeleton bears oxygen-containing substituents at both C-2 and C-3. The HMBC correlations of the acetyl *C*O signal with H-3 and the acetyl methyl signal suggested the presence of an acetate group at C-3, while, based on the HMBC correlations of H-2/C-1′, H-2′/C-1′, H-2′/C-3′, H-2′/C-4′, H-2′/C-5′, H-4′/C-5′, and H-5′/C-6′, a 3-hydroxy-3-methylglutarate moiety was attached to C-2 in compounds **1** and **2**. The additional H-7′/C-6′ HMBC correlation in compound **2** suggested that **2** was the methyl ester of compound **1**. Further to these, the HMBC correlations of the doublet of doublets at δ_H_ 1.88/1.85 (assigned to H-5) with C-4, C-10, C-19, C-29, and C-30 confirmed this assignment, while long-range correlations of H-5 with the methylene at δ_C_ 36.2/36.2 and with the carbonyl at δ_C_ 197.8/197.8 enabled the assignment of C-6 and suggested the presence of a keto group in position 7. In parallel, the HMBC correlations between the diastereotopic protons (H-22) and the methylene group at δ_C_ 32.6/32.5 (C-23) and carbonyl group at δ_C_ 214.9/214.8 (C-24) and between the methyl singlets of H-26 and H-27 and C-24 and nonprotonated carbon at δ_C_ 76.2/76.2 (C-25) led to the conclusion that a 24-keto-25-hydroxy side chain was present at C-17. Based on the complete ^1^H and ^13^C NMR assignments (Tables 1 and 2), compounds **1** and **2** were identified as fasciculic acid A^11,12^ derivatives.

**Table 1:** ^1^H NMR Data (800 MHz) of Compounds **1**–**4** in CDCl_3_

	δ_H_, mult (*J* in Hz)			
atom#	**1**	**2**	**3**	**4**
1β	2.25, dd (12.2, 4.1)	2.22, dd (12.3, 4.3)	2.12, dd (12.1, 4.2)	2.15, dd (12.2, 4.4)
1α	1.57, t (12.2)	1.54, t (12.3)	1.37, m	1.40, m
2	5.22, td (10.8, 4.1)	5.20, td (10.8, 4.3)	5.17, td (10.8, 4.2)	5.18, td (10.8, 4.4)
3	4.85, d (10.8)	4.83, d (10.8)	4.79, d (10.8)	4.81, d (10.8)
5	1.88, dd (13.3, 4.0)	1.85, dd (13.3, 4.0)	1.26, m	1.30, m
6β	2.46, m (2H)	2.44, m (2H)	1.55, m	1.57, m
6α			1.70, m	1.72, m
7			2.06, m	2.06, m
11β	2.31, m (2H)	2.30, m (2H)	1.78, m	1.79, m
11α			2.49, m	2.52, m
12	1.81, m	1.79, m	4.13, t (7.7)	4.14, t (7.7)
15β	1.75, m	1.74, m	1.70, m	1.73, m
15α	2.10, m	2.07, m	1.21, m	1.25, m
16β	1.41, m	1.40, m	1.56, m	1.58, m
16α	2.01, m	2.01, m	1.89, m	1.91, m
17	1.47, m	1.44, m	1.87, m	1.89, m
18	0.67, s	0.65, s	0.72, s	0.74, s
19	1.32, s	1.30, m	1.14, s	1.16, s
20	1.43, m	1.40, m	1.66, m	1.67, m
21	0.93, d (6.6)	0.92, d (6.6)	1.01, d (6.6)	1.04, d (6.6)
22	1.85, m	1.83, m	1.28, m	1.31, m
22	1.33, m	1.31, m	1.92, m	1.95, m
23	2.59, m	2.57, ddd (17.1, 9.8, 5.1)	2.56, m	2.60, dd (9.5, 5.4)
23	2.51, m	2.49, ddd (17.1, 9.3, 6.1)		
26	1.41, s	1.38, s	1.39, s	1.41, s
27	1.41, s	1.39, s	1.38, s	1.40, s
28	0.94, s	0.92, m	0.90, s	0.93, s
29	1.03, s	1.00, s	0.94, s	0.95, s
30	0.93, s	0.91, m	0.91, s	0.93, m
2′	2.57, m	2.54, AB (15.4)	2.53, m	2.56, AB (15.6)
2′	2.70, m	2.68, AB (15.4)	2.68, m	2.70, AB (15.6)
4′	1.40, s	1.35, s	1.38, s	1.36, s
5′a	2.55, br m	2.62, AB (15.4)	2.64, m	2.73, AB (14.9)
5′b	2.72, br m	2.71, AB (15.4)	2.68, m	2.64, AB (14.9)
7′		3.71, s		3.73, s
3-OAc	2.09, s	2.07, s	2.06, m	2.08, s
25-OH		3.82, s		3.83, s
3′-OH		3.91, s		3.96, s

**Table 2:** ^13^C NMR Data (200 MHz) of Compounds **1**–**4** in CDCl_3_

	δ_C_, type			
atom#	**1**	**2**	**3**	**4**
1	40.1, CH_2_	40.1, CH_2_	41.0, CH_2_	41.1, CH_2_
2	70.3, CH	69.9, CH	71.2, CH	70.7, CH
3	79.0, CH	78.9, CH	80.1, CH	80.0, CH
4	39.3, C	39.2, C	39.3, C	39.3, C
5	49.3, CH	49.3, CH	49.9, CH	50.0, CH
6	36.2, CH_2_	36.2, CH_2_	17.9, CH_2_	17.9, CH_2_
7	197.8, C	197.8, C	25.8, CH_2_	25.9, CH_2_
8	139.2, C	139.1, C	134.8, C	134.7, C
9	163.1, C	163.0, C	134.2, C	134.2, C
10	40.5, C	40.5, C	37.8, C	37.9, C
11	23.8, CH_2_	23.8, CH_2_	33.8, CH_2_	34.0, CH_2_
12	29.9, CH_2_	29.9, CH_2_	72.8, CH_2_	72.8, CH_2_
13	44.9, C	44.9, C	49.0, C	49.0, C
14	47.8, C	47.8, C	52.2, C	52.2, C
15	31.8, CH_2_	31.8, CH_2_	31.2, CH_2_	31.1, CH_2_
16	28.6, CH_2_	28.6, CH_2_	25.1, CH_2_	25.2, CH_2_
17	48.9, CH	48.9, CH	50.5, CH	50.6, CH
18	15.8, CH_3_	15.7, CH_3_	9.9, CH_3_	9.8, CH_3_
19	19.4, CH_3_	19.4, CH_3_	20.0, CH_3_	20.0, CH_3_
20	35.9, CH	35.9, CH	33.8, CH	33.8, CH
21	18.5, CH_3_	18.5, CH_3_	21.2, CH_3_	21.2, CH_3_
22	30.1, CH_2_	30.0, CH_2_	29.1, CH_2_	29.1, CH_2_
23	32.6, CH_2_	32.5, CH_2_	33.7, CH_2_	33.6, CH_2_
24	214.9, C	214.8, C	215.2, C	215.1, C
25	76.2, C	76.2, C	76.3, C	76.2, C
26	26.5, CH_3_	26.6, CH_3_	26.5, CH_3_	26.5, CH_3_
27	26.5, CH_3_	26.5, CH_3_	26.5, CH_3_	26.5, CH_3_
28	25.0, CH_3_	25.0, CH_3_	24.2, CH_3_	24.1, CH_3_
29	17.3, CH_3_	17.2, CH_3_	17.4, CH_3_	17.4, CH_3_
30	27.6, CH_3_	27.6, CH_3_	28.2, CH_3_	28.2, CH_3_
1′	170.8, C	171.2, C	171.5, C	171.4, C
2′	44.9, CH_2_	44.8, CH_2_	44.7, CH_2_	44.9, CH_2_
3′	69.6, C	69.5, C	69.7, C	69.5, C
4′	27.1, CH_3_	27.3, CH_3_	27.2, CH_3_	26.5, CH_3_
5′	44.8, CH_2_	44.7, CH_2_	45.0, CH_2_	44.7, CH_2_
6′	173.7, C	172.1, C	173.5, C	172.1, C
7′		51.7, CH_3_		51.7, CH_3_
3-OAc	171.4, C	170.6, C	171.1, C	170.8, C
	20.9, CH_3_	20.9, CH_3_	21.0, CH_3_	21.0, CH_3_

According to the observed NOE correlations of H-18 with H-11β, H-15β, H-16β, and H-20, those of H-19 with H-1β, H-2, H-11β, and H-29, and of H-28 with H-15α, H-16α and H-17, the relative stereochemistry as depicted in structural formulas **1** and **2** was proposed for the two compounds. Thus, compound **1**, named as pholiol A, was assigned as the 3-*O*-acetyl-7,24-diketo analogue of fasciculic acid A,^11,12^ while pholiol B (**2**) was assigned as its methyl ester. The relative configuration of the C-3′ chiral center could not be determined on this basis and is only tentatively given as *S*, based on the close chemical shift values with those of similar compounds^11,12^ and on assuming that similar metabolic pathways led to the formation of the structurally similar compounds in the different fungal species.

The molecular formulas of **3** (C_38_H_60_O_10_) and **4** (C_39_H_62_O_10_) derived from HRESIMS measurements differed only in a methylene group, similarly to those of compound pair **1** and **2**. As compared to pholiols A (**1**) and B (**2**), compounds **3** and **4** contained two additional hydrogens, nominally corresponding to the saturation of the double bond in **1**/**2**. The ^1^H and ^13^C NMR spectra of **3** and **4** were highly similar to those of compounds **1** and **2**. The most striking differences were the presence of a triplet signal with one hydrogen intensity at δ_H_ 4.13/4.14 in the ^1^H NMR spectrum and in parallel the absence of the carbonyl resonance belonging to C-7 and the appearance of resonance at δ_C_ 72.8/72.8 in the ^13^C NMR spectrum of **3**/**4**. Thus, at first sight, the reduction of the keto group at C-7 to a hydroxy group was envisaged. The ^1^H and ^13^C NMR spectra of **3** and **4** were almost identical, except for the presence of a singlet with a three-hydrogen intensity at δ_H_ 3.73 in the ^1^H NMR spectrum and a carbon signal at δ_C_ 51.7 in the ^13^C NMR spectrum of **4**. This suggested that **4** was the methyl ester analogue of compound **3**. Analysis of the HSQC and HMBC data showed that a hydroxy group was attached to C-12 (δ_H-12_ 4.13/4.14, each 1H, t, *J* = 7.7 Hz; δ_C-12_ 72.8/72.8 for **3**/**4**; HMBC correlation between H-18 and C-12) and a methylene group was present in position 7 based on the HMBC correlation of H-5 and H-6 with C-7. 2D NMR experiments allowed the complete ^1^H and ^13^C NMR assignments of **3** and **4** as listed in Tables 1 and 2. Compound **3**, named as pholiol C, was identified as the 24-keto derivative of fasciculic acid B,^11^ while pholiol D (**4**) was identified as its methyl ester. The similar chemical shifts and NOE correlations suggested that the relative configurations of the C-2, C-3, C-5, C-10, C-14, C-17, and C-20 (and C-1′) chiral centers of **3** and **4** were identical to those determined for pholiols A (**1**) and B (**2**). The NOE correlations of H-12 with H-11α, H-17, and H-28 (Figure S26) suggested that the hydroxy group occupied the β position (thus, the C-12 chiral center had an *R* configuration) in pholiols C (**3**) and D (**4**).

Compound **5** was identified as (6*E*,10*E*,14*E*,18*E*)-2,3,22,23-tetrahydroxy-2,6,10,15,19,23-hexamethyl-6,10,14,18-tetracosatetraene regarding its ^1^H and ^13^C NMR chemical shift values, identical with literature data.^13^ The 3*S*,22*S* configuration of **5** can be suggested based on the opposite optical rotation data than that of the 3*R*,22*R* isomer.^14^ Compound **6** was found to be identical in all of its spectroscopic characteristics with that of ergosterol.^15^ 3β-Hydroxyergosta-7,22-diene was detected in the *n*-hexane fraction with the use of an authentic standard.

### Cytotoxic Activity

The isolated compounds (**1**–**6**) were tested for their cytotoxic activity on sensitive Colo 205 and resistant Colo 320 cell lines and on the normal MRC-5 embryonal fibroblast cell line using the 3-(4,5-dimethylthiazol-2-yl)-2,5-diphenyltetrazolium bromide (MTT) assay with doxorubicin as a positive control. Among the studied compounds, ergosterol (**6**) showed substantial cytotoxic activity against the tumor cell lines with IC_50_ values of 4.9 μM (Colo 205) and 6.5 μM (Colo320) (Table 3). This compound was more potent against the MRC-5 cell line (IC_50_ 0.50 μM). Pholiols B (**2**) and D (**4**) and compound **5** possessed weak inhibitory activities (IC_50_ > 25 μM) against the tested cell lines without any selectivity (Table S2).

**Table 3:** Cytotoxic Effect of the Compound **6**

	Colo205 (IC_50_ μM)		Colo320 (IC_50_ μM)		MRC-5 (IC_50_ μM)	
compound	mean	SD	mean	SD	mean	SD
ergosterol (**6**)	**4.9**	0.6	**6.5**	0.2	**0.5**	0.1
doxorubicin	2.5	0.3	7.4	0.2	>20	

### MDR Efflux Pump Inhibitory Activity

The inhibitory activities of compounds **1**–**3**, **5**, and **6** on efflux function were evaluated by measuring the intracellular accumulation of rhodamine 123, a well-known P-glycoprotein substrate fluorescent dye, within the Colo320 MDR cells. Tariquidar, a strong P-gp inhibitor, was used as positive control. All tested compounds were dissolved in DMSO, and the final concentration (2.00%) of the solvent was investigated for any effect on the retention of rhodamine 123. The results revealed inhibition of P-gp MDR efflux pump activity manifested by pholiols A (**1**) and B (**2**) and triterpene **5**. In general, compounds with fluorescence activity ratio (FAR) values greater than 1 were considered to be active P-gp inhibitors, while compounds with FAR values greater than 10 were considered to be strong MDR modulators. The sterol compounds with methyl ester functionality (**2**) and the polyhydroxy-squalene derivative (**5**) exerted the highest anti-MDR effect in this bioassay with FAR values of 6.880 and 6.638, respectively.

**Table 4:** P-gp Efflux Pump Inhibitory Activity of Compounds **1**–**3**, **5**, and **6** against MDR COLO 320 Colon Adenocarcinoma Cells

sample	conc., μM	FAR
tariquidar^a^	0.2	5.533
pholiol A (**1**)	20	3.418
pholiol B (**2**)	20	**6.880**
pholiol C (**3**)	20	0.967
compound **5**	20	**6.638**
ergosterol (**6**)	2	1.053
DMSO	2.00%	0.828

a Positive control.

### Combination Studies with Doxorubicin

Compounds **2** and **4**–**6** were tested for their capacity to reduce the resistance of the MDR Colo 320 cell line to doxorubicin. A checkerboard microplate combination assay was performed, which is a widely used *in vitro* method for the assessment of drug interactions. Experimental data were analyzed using CompuSyn software, which enabled the determination of the most effective ratios of combined agents and calculation of combination indices (CI). Based on the combination indices, the type of interaction could be defined according to the literature.^16^ Pholiols B (**2**) and D (**4**), with a 3-hydroxy-3-methyl-glutarate methyl ester moiety, and **5** interacted in an synergistic manner, and CI values at 50% of the ED_50_ were found to be 0.348, 0.660, and 0.082, respectively. The outstanding potency of **5**, designated as very strong synergism (CI = 0.082), is promising. In this assay ergosterol (**6**) was found to have an additive effect in combination with doxorubicin.

It is noteworthy that the tetrahydroxy-squlene derivative (**5**) has the capacity to potentiate the effect of doxorubicin in Colo 320 adenocarcinoma cells by P-gp modulation and strong synergism with doxorubicin, and therefore it represents a promising new class of potential adjuvants of cancer chemotherapy. With regard to the moderate P-gp inhibitory activity and strong synergism of **5** in combination with doxorubicin, the mechanism of its chemosensitizing effect should have another P-gp-independent mechanism.

**Table 5:** Chemosensitizing Activity of Compounds **2** and **4**–**6** on Colo 320 Adenocarcinoma Cells

compound	best ratio^a^	CI at ED_50_^b^	SD	interaction
pholiol B (**2**)	23.2:1	0.348	0.051	synergism
pholiol D (**4**)	139.2:1	0.660	0.03	Synergism
compound **5**	2.9:1	0.082	0.057	very strong synergism
ergosterol (**6**)	3.6:1	1.03	0.12	nearly additive

a Best ratio: the best combination ratio between compound and doxorubicin.

b CI at ED_50_: combination index value at the 50% growth inhibition dose.

### Antibacterial Effect

Compounds **1**–**6** were inactive against *Escherichia coli* ATCC 25922, *Salmonella enterica* serovar Typhimurium 14028s, *Staphylococcus aureus* ATCC 25923, and *S. aureus* 27213.^8^

## Experimental Section

### General Experimental Procedures

The chemicals used in the experiments were supplied by Sigma-Aldrich Hungary and Molar Chemicals, Hungary. An InsMark IP-Digi1 polarimeter (Shanghai InsMark Instrument Technology Co., Ltd., Shanghai, China) was applied for optical rotation measurements. Flash chromatography (FC) was carried out on a CombiFlash Rf+ Lumen Instrument with integrated UV, UV–vis, and ELS detection using reversed (RediSep C_18_ Bulk 950) (Teledyne Isco, Lincoln, NE, USA) and normal phase flash columns filled with silica 60 (0.045–0.063 mm) (Molar Chemicals, Halásztelek, Hungary) and also RediSep Rf Gold normal (Teledyne Isco). Preparative thin layer chromatography (TLC) was performed using silica plates (20 × 20 cm silica gel 60 F254, Merck 105554). High-resolution MS (HRMS) analyses were performed on a Thermo Velos Pro Orbitrap Elite (Thermo Fisher Scientific, Bremen, Germany) system. The ionization method was ESI, operating in the positive (or in the negative) ion mode. The (de)protonated molecular ion peaks were fragmented by the collision-induced dissociation method (CID) at a normalized collision energy of 35%. For the CID experiments, helium was used as collision gas. All samples were dissolved in methanol prior to the MS measurements. Data acquisition and analysis were accomplished with Xcalibur software version 4.0 (Thermo Fisher Scientific, Bremen, Germany). NMR data were acquired on a Bruker Avance III HD 800 MHz spectrometer equipped with a liquid helium cooled TCI cryoprobe. CDCl_3_ was used as solvent in all cases. Chemical shifts are reported in the delta scale relative to a tetramethylsilane internal standard (0.00 ppm, 1H) and to the residual solvent signal (77.0 ppm, 13C). Standard one- and two-dimensional NMR spectra were recorded in all cases using the pulse sequences available in the TopSpin 3.5 sequence library. Data analysis and interpretation were performed with the ACD/Laboratories 2017.1.3 NMR Workbook Suite package.

### Mushroom Material

Fruiting bodies of *Pholiota populnea* were collected in autumn 2017 in the vicinity of Szeged, Hungary. Fungal identification was made by Attila Sándor (Mushroom Society of Szeged). A voucher specimen (No. H019) has been deposited at the Department of Pharmacognosy, University of Szeged, Hungary.

### Extraction and Isolation

The fresh mushroom material (4.2 kg) was crushed in a blender and then percolated with MeOH (20 L) at room temperature. After concentration, the dry MeOH extract (151 g) was dissolved in 50% aqueous MeOH (600 mL) and solvent–solvent partition was performed with *n*-hexane (5 × 500 mL), CHCl_3_ (5 × 500 mL), and then EtOAc (5 × 500 mL). The *n*-hexane-soluble phase (24 g) was subjected to flash chromatography (NP-FC) on silica gel (80 g) using a gradient system of *n*-hexane–acetone (linear from 0% to 100% acetone, *t* = 55 min) and eluted with MeOH (100%, *t* = 5 min) at the end of this process. Fractions with similar compositions were combined according to TLC monitoring (H1–H26). Fractions H20 (130 mg), H21 (400 mg), and H22 (200 mg) were further separated by NP-FC (12 g of sorbent) applying an *n*-hexane–acetone solvent system (linear gradient from 0% to 30% acetone, *t* = 50 min) and then using *n*-hexane–acetone (linear gradient from 0% to 35% acetone, *t* = 40 min), which led to the isolation of compound **5** (10 mg) and obtaining other 13 fractions (D1–D13). Purification of D9 and D10 fractions was performed by preparative TLC using an *n*-hexane–acetone (45:55) solvent system to give compound **4** (13 mg). Fractions H23 and H24 (1.7 g) were subjected to NP-FC (20 g sorbent) using a solvent system of *n*-hexane–acetone (linear gradient from 0% to 80% acetone, *t* = 50 min), which resulted in 22 fractions (B1–B22). The combined fractions B16 (94 mg) and B17 (294 mg) were purified by NP-FC (12 g of sorbent) using a mixture of *n*-hexane–acetone (linear gradient from 0% to 50% acetone, *t* = 55 min) to obtain compound **1** (18 mg) and compound **3** (12 mg), respectively. Fractions B10 (95 mg) and B11 (300 mg) were first separated by FC on an RP_18_ column (20 g) using H_2_O–MeOH (linear gradient from 60% to 90% MeOH, *t* = 50 min), then by preparative TLC using CHCl_3_–MeOH (95:5) to isolate compound **2**. Finally, compound **6** (130 mg) was isolated from fractions H10–H12 (2 g) using *n*-hexane–acetone (40 g of sorbent, linear gradient from 100:0 to 75:25, *t* = 45 min). The presence of 3β-hydroxyergosta-7,22-diene (**7**) was detected in the *n*-hexane fraction using an authentic standard.

#### Pholiol A (**1**):

amorphous solid; [α]_D_ +10 (*c* 0.1, CHCl_3_); HRESIMS *m*/*z* 673.39752 [M – H]^−^ (Δ 2.7 ppm; C_38_H_57_O_10_); ^1^H and ^13^C NMR data, see Tables 1 and 2; HRESI-MSMS (CID = 35%; rel int %) *m*/*z* 611(100), 571(63), 529(44), 469(4).

#### Pholiol B (**2**):

amorphous solid; [α]_D_ +80 (*c* 0.1, CHCl_3_); HRESIMS *m*/*z* 689.42289 [M + H]^+^ (Δ −4.4 ppm, C_39_H_61_O_10_); ^1^H and ^13^C NMR data, see Tables 1 and 2; HRESI-MSMS (CID = 35%; rel int %) *m*/*z* 671(8), 531(100), 513(30), 471(55), 453(56), 435(8), 417(4), 377(1).

#### Pholiol C (**3**):

amorphous solid; [α]_D_ +16 (*c* 0.1, CHCl_3_); HRESIMS *m*/*z* 675.41283 [M – H]^−^ (Δ 2.2 ppm, C_38_H_59_O_10_).; ^1^H and ^13^C NMR data, see Tables 1 and 2; HRESI-MSMS (CID = 35%; rel int %) *m*/*z* 613(100), 573(92), 555(6), 531(31).

#### Pholiol D (**4**):

white powder; [α]_D_ −90 (*c* 0.1, CHCl_3_); HRESIMS *m*/*z* 1403.85130 [2M + Na]^+^ (Δ −4.6 ppm, C_78_H_124_O_20_Na); ^1^H and ^13^C NMR data, see Tables 1 and 2; HRESI-MS^3^ (1408/713; CID = 35%, 45%; rel int %) *m*/*z* 653(12), 639(6), 537(100), 477(41), 437(3).

#### (3*S*,6*E*,10*E*,14*E*,18*E*,22*S*)-2,3,22,23-Tetrahydroxy-2,6,10,15,19,23-hexamethyl-6,10,14,18-tetracosatetraene (**5**):

amorphous solid; [α]_D_ −2.3 (*c* 0.16, CHCl_3_) and −15.75 (*c* 0.16, MeOH); ^1^H and ^13^C NMR data, Table S1; HRESIMS *m*/*z* 479.40900 [M + H]^+^ (Δ −1.0 ppm; C_30_H_55_O_5_); HRESI-MSMS (CID = 35%; rel int %) *m*/*z* 461(100), 443(31).

### Cell Culture

The human colon adenocarcinoma cell lines, the Colo 205 (ATCC-CCL-222) doxorubicin-sensitive parent and Colo 320/MDR-LRP (ATCC-CCL-220.1) resistant to anticancer agents expressing ABCB1, were purchased from LGC Promochem (Teddington, UK). The cells were cultured in RPMI-1640 medium supplemented with 10% heat-inactivated fetal bovine serum (FBS), 2 mM l-glutamine, 1 mM Na-pyruvate, 100 mM Hepes, nystatin, and a penicillin–streptomycin mixture in concentrations of 100 U/L and 10 mg/L, respectively. The MRC-5 (ATCC CCL-171) human embryonic lung fibroblast cell line (LGC Promochem) was cultured in EMEM medium, supplemented with 1% nonessential amino acid mixture, 10% heat-inactivated FBS, 2 mM l-glutamine, 1 mM Na-pyruvate, nystatin, and a penicillin–streptomycin mixture in concentrations of 100 U/L and 10 mg/L, respectively. The cell lines were incubated in a humidified atmosphere (5% CO_2_, 95% air) at 37 °C.

### Assay for Cytotoxic Effect

The effects of increasing concentrations of the compounds on cell growth were tested in 96-well flat-bottomed microtiter plates. The 2-fold serial dilutions of the tested compounds were made starting with 100 μM. Then, 10^4^ human colonic adenocarcinoma cells in 100 μL of the medium (RPMI-1640) were added to each well, except for the medium control wells. The adherent human embryonic lung fibroblast cell line (10^4^/well) was seeded in EMEM medium in 96-well flat-bottomed microtiter plates for 4 h before the assay. The serial dilutions of the compounds were made in a separate plate starting with 100 μM and then transferred to the plates containing the adherent corresponding cell line. Culture plates were incubated at 37 °C for 24 h; at the end of the incubation period, 20 μL of MTT (thiazolyl blue tetrazolium bromide) solution (from a 5 mg/mL stock solution) was added to each well. After incubation at 37 °C for 4 h, 100 μL of sodium dodecyl sulfate (SDS) solution (10% SDS in 0.01 M HCl) was added to each well, and the plates were further incubated at 37 °C overnight. Cell growth was determined by measuring the optical density (OD) at 540 nm (ref 630 nm) with a Multiscan EX ELISA reader (Thermo Labsystems, Cheshire, WA, USA). Inhibition of cell growth was expressed as IC_50_ values, defined as the inhibitory dose that reduces the growth of the cells exposed to the tested compounds by 50%. IC_50_ values and the SD of triplicate experiments were calculated by using GraphPad Prism software version 5.00 for Windows with nonlinear regression curve fit (GraphPad Software, San Diego, CA, USA; www.graphpad.com).

### Rhodamine 123 Accumulation Assay

The cell numbers of the human colon adenocarcinoma cell lines were adjusted to 2 × 10^6^ cells/mL, resuspended in serum-free RPMI 1640 medium, and distributed in 0.5 mL aliquots into Eppendorf centrifuge tubes. The tested compounds were added at 2 or 20 μM concentrations, and the samples were incubated for 10 min at room temperature. Tariquidar was applied as positive control at 0.2 μM. DMSO at 2% v/v was used as solvent control. Next, 10 μL (5.2 μM final concentration) of the fluorochrome and ABCB1 substrate rhodamine 123 (Sigma) were added to the samples, and the cells were incubated for a further 20 min at 37 °C, washed twice, and resuspended in 1 mL of PBS for analysis. The fluorescence of the cell population was measured with a PartecCyFlow flow cytometer (Partec, Münster, Germany). The FAR was calculated as the quotient between FL-1 of the treated/untreated resistant Colo 320 cell line over the treated/untreated sensitive Colo 205 cell line according to the following equation:
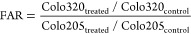


### Checkerboard Combination Assay

A checkerboard microplate method was applied to study the effect of drug interactions between the compounds (**2**, **4**–**6**) and the chemotherapeutic drug doxorubicin. The assay was carried out on the Colo 320 colon adenocarcinoma cell line. The final concentration of the compounds and doxorubicin used in the combination experiment was chosen in accordance with their cytotoxicity toward this cell line. The dilutions of doxorubicin were made in a horizontal direction in 100 μL, and the dilutions of the compounds vertically in the microtiter plate in a 50 μL volume. Then, 6 × 10^3^ of Colo 320 cells in 50 μL of the medium were added to each well, except for the medium control wells. The plates were incubated for 72 h at 37 °C in a 5% CO_2_ atmosphere. The cell growth rate was determined after MTT staining. At the end of the incubation period, 20 μL of MTT solution (from a stock solution of 5 mg/mL) was added to each well. After incubation at 37 °C for 4 h, 100 μL of SDS solution (10% in 0.01 M HCI) was added to each well, and the plates were further incubated at 37 °C overnight. OD was measured at 540 nm (ref 630 nm) with a Multiscan EX ELISA reader. CI values at 50% of the growth inhibition dose were determined using CompuSyn software (ComboSyn, Inc., Paramus, NJ, USA) to plot four to five data points at each ratio. CI values were calculated by means of the median-effect equation, according to the Chou–Talalay method, where CI < 1, CI = 1, and CI > 1 represent synergism, additive effect (or no interaction), and antagonism, respectively.^17,18^

### Bacterial Strains and Determination of Antibacterial Activity

See ref (8).

## Supporting Information Available

1D and 2D NMR and HRESIMS spectra of compounds **1**–**5** (PDF)
