# Supplementary material for: Triterpenes from Pholiota populnea as Cytotoxic Agents and Chemosensitizers to Overcome Multidrug Resistance of Cancer Cells
Source: J Nat Prod. 2022 Mar 16;85(4):910–6. doi: 10.1021/acs.jnatprod.1c01024 (PMC9040055; doi:10.1021/acs.jnatprod.1c01024)
Supplement: Supplementary file 1 — np1c01024_si_001.pdf [file np1c01024_si_001.pdf]

Triterpenes from *Pholiota populnea* as Cytotoxic Agents and Chemosensitizers to Overcome Multidrug Resistance of Cancer Cells

Morteza Yazdani,[†] Zoltán Béni,[‡] Miklós Dékány,[‡] Nikoletta Szemerédi,[§] Gabriella Spengler,[§] Judit Hohmann,[†,^,*] and Attila Ványolós[‖,*]

[†] *Department of Pharmacognosy, Interdisciplinary Excellence Centre, University of Szeged, 6720 Szeged, Hungary*

[‡] *Spectroscopic Research Department, Gedeon Richter Plc., Gyömrői út 19-21, H-1103 Budapest, Hungary*

[§] *Department of Medical Microbiology, Albert Szent-Györgyi Health Center and Faculty of Medicine, University of Szeged, Semmelweis utca 6, H-6725 Szeged, Hungary*

[^] *Interdisciplinary Centre for Natural Products, University of Szeged, Eötvös u. 6, H-6720 Szeged, Hungary*

[‖] *Department of Pharmacognosy, Semmelweis University, Üllői u. 26, H-1085 Budapest, Hungary*

*****Correspondence: hohmann.judit@szte.hu (J.H.). Tel.: +36-62-546453

TABLE OF CONTENTS

Figure S1. HRESI-MS spectrum of compound **1** ... 4

Figure S2. MSMS spectrum of compound **1** ... 5

Figure S3. HRMS spectrum of compound **2** ... 6

Figure S4. MSMS spectrum of compound **2** ... 6

Figure S5. ^{1}H and ^{13}C spectra of compound **1** ... 7

Figure S6. HMBC spectrum of compound **1** ... 8

Figure S7. HSQC spectrum of compound **1** ... 9

Figure S8. 1D ROESY spectrum of compound **1** ... 10

Figure S9. 1D ROESY spectrum of compound **1** ... 10

Figure S10. 1D ROESY spectrum of compound **1** ... 11

Figure S11. 1D ROESY spectrum of compound **1** ... 11

Figure S12. 1D ROESY spectrum of comppound **1** ... 12

Figure S12. 1D ROESY spectrum of compound **1** ... 12

Figure S13. ^{1}H NMR spectra of compounds **1** and **3** ... 13

Figure S14. ^{13}C NMR spectra of compounds **1** and **2**... 13

Figure S15. COSY spectrum of compound **2** ... 14

Figure S16. HMBC spectrum of compound **2** ... 14

Figure S17. HSQC spectrum of compound **2** ... 15

Figure S18. ROESY spectrum of compound **2** ... 15

Spectra and spectral data on compounds 3 and 4 ... 16

Figure S19. HRMS spectra of compound **3** ... 16

Figure S20. HRMS-MS spectra of compound **3** ... 17

Figure S21. 800 MHz ^{1}H NMR spectrum of compound **3** ... 18

Figure S22. 200 MHz ^{13}C NMR spectrum of compound **3** ... 18

Figure S23. 800 MHz HMBC spectrum of compound **3** ... 19

Figure S24. 800 MHz HSQC NMR spectrum of compound 3 ... 19

Figure S25. 1D ROESY spectrum of compound **3** ... 20

Figure S26. 1D ROESY spectrum of compound **3** ... 20

Figure S27. 1D ROESY spectrum of compound **3** ... 21

Figure S28. HRMS spectra of compound **4** ... 22

Figure S29. HRMS-MS spectra of compound **4** ... 23

Figure S30. 800 MHz ^{1}H NMR spectra of compounds **3** (top) and **4** (bottom) ... 24

Figure S31. 200 MHz ^{13}C NMR spectra of compounds **3** (top) and **4** (bottom) ... 24

Figure S32. HSQC spectrum of compound **4** 25
Figure S33. HMBC spectrum of compound **4** 25
Figure S34. ESI-HR-MS spectrum of compound **5** 26
Figure S35. ESI-HR-MS-MS of compound **5** 27
Figure S36. ^{1}H NMR spectrum of compound **5**..... 28
Figure S37. ^{13}C NMR spectrum of compound **5**..... 28
Table S1. ^{1}H and ^{13}C NMR assignments of compound **5**..... 29
Table S2. Cytotoxic effect of the compounds (**1**-**6**) isolated from *H. populnea*..... 29
Table S3. P-gp Efflux Pump Inhibitory Activity of Compounds **1**-**3**, **5** and **6** against MDR COLO 320 Colon Adenocarcinoma Cells..... 30

Spectra and spectral data on compounds 1 and 2

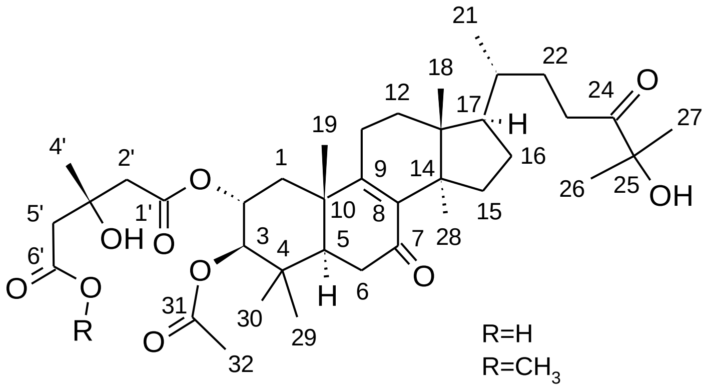

Compound **1**

HRMS: M-H=673.39752 (delta=2.7 ppm; $C_{38}H_{57}O_{10}$). HR-ESI-MS-MS (CID=35%; rel. int. %): 611(100); 571(63); 529(44); 469(4).

^{1}H and ^{13}C NMR assignments are given in Table 1

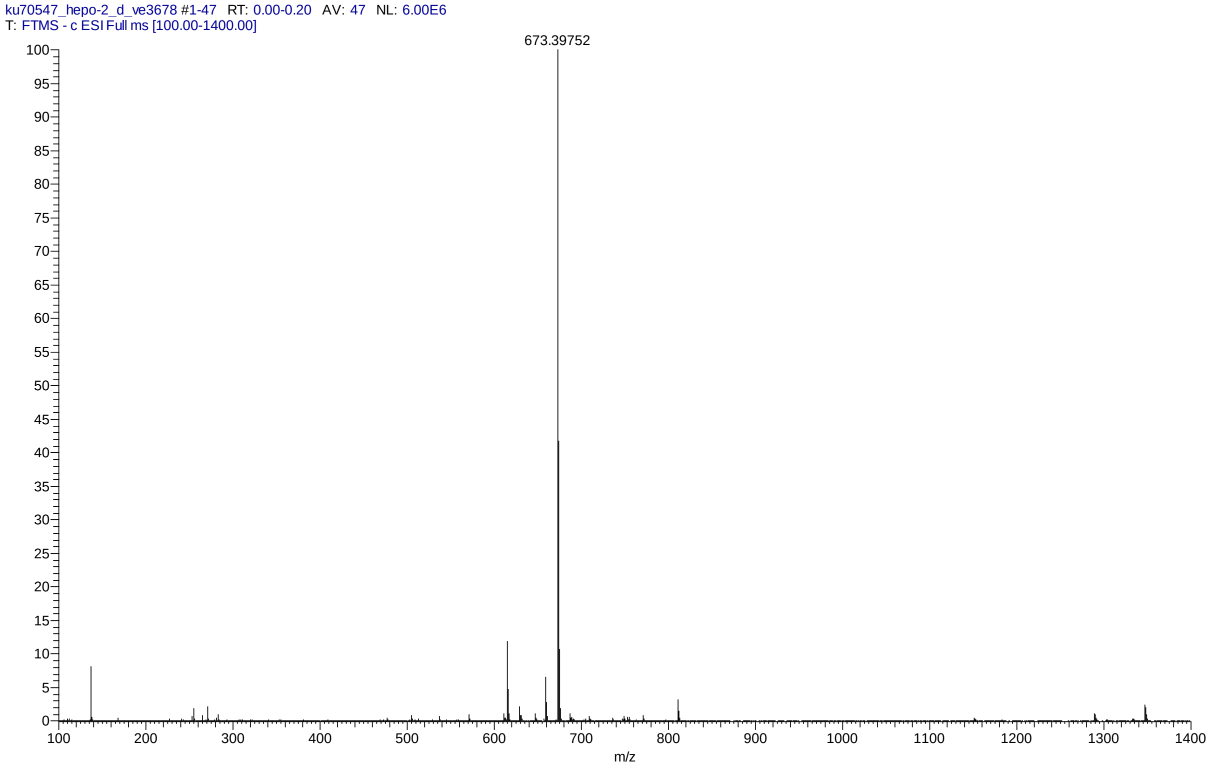

Figure S1. HRESI-MS spectrum of compound **1**

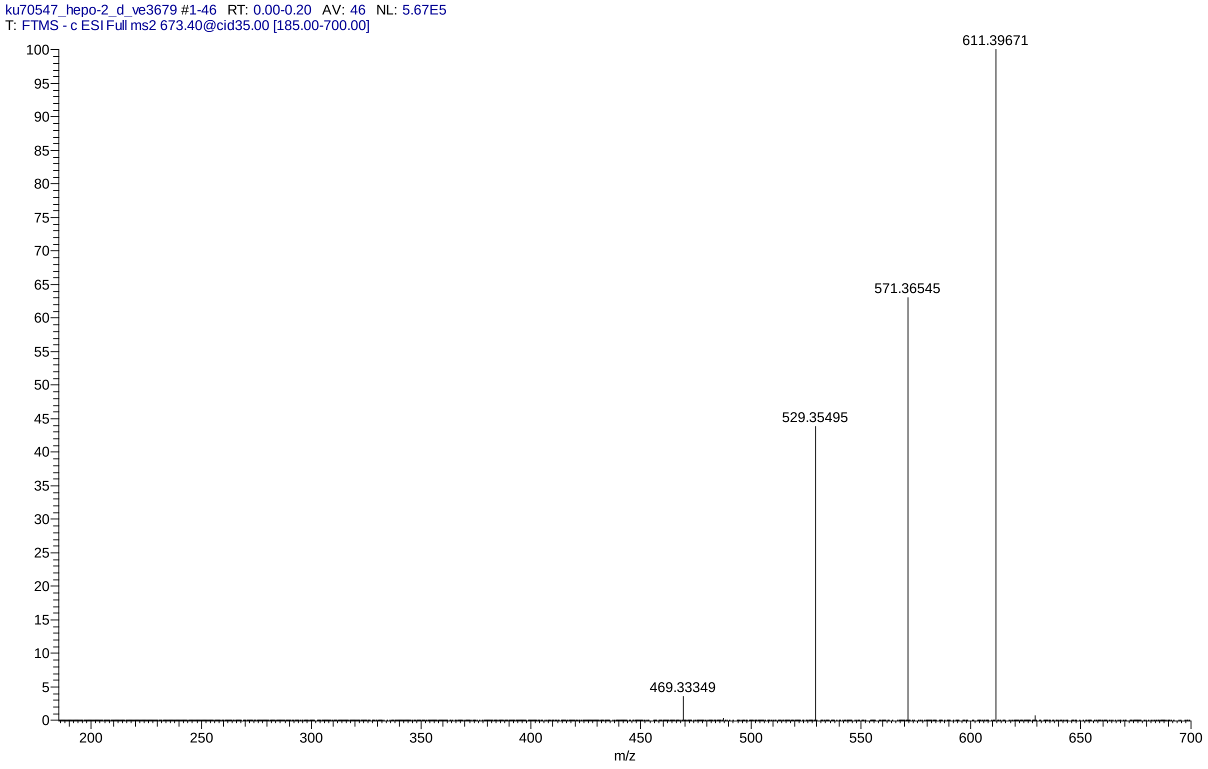

Figure S2. MSMS spectrum of compound **1**

Compound **2**

HRMS: M+H=689.42289 (delta=-4.4 ppm; $C_{39}H_{61}O_{10}$). HR-ESI-MS-MS (CID=35%; rel. int. %): 671(8); 531(100); 513(30); 471(55); 453(56); 435(8); 417(4); 377(1).

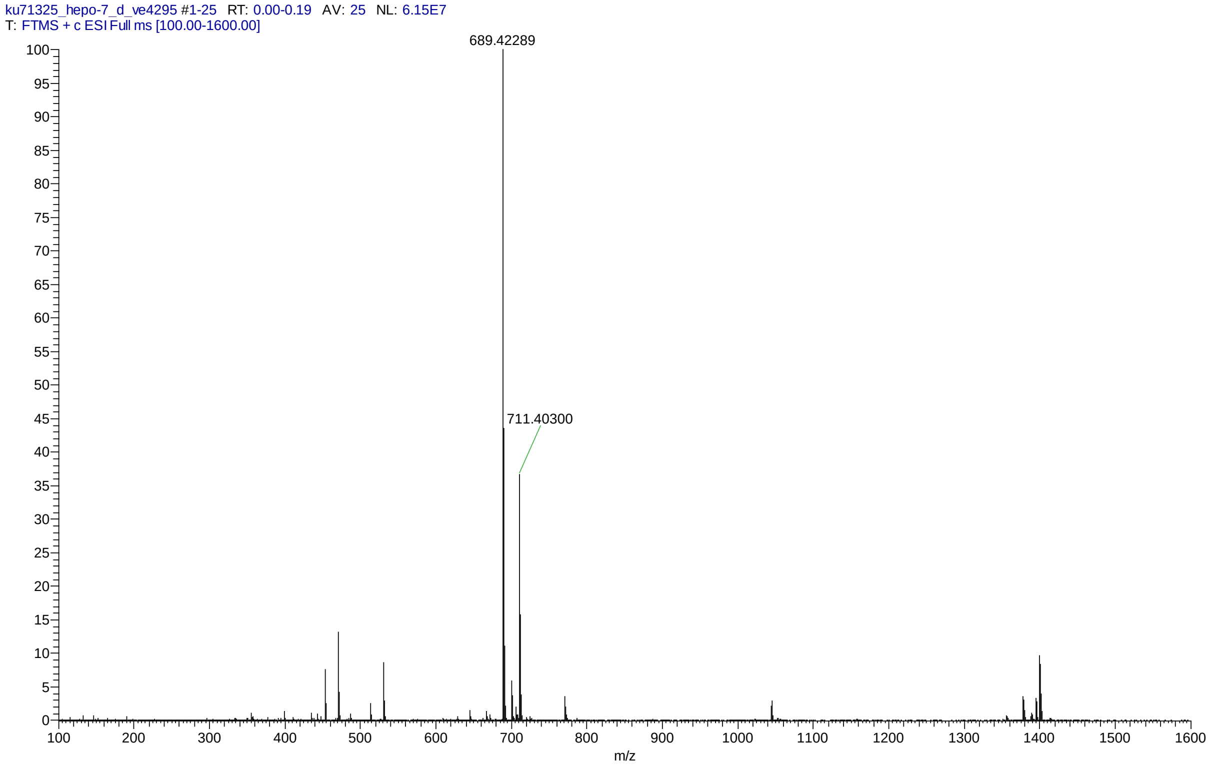

Figure S3. HRMS spectrum of compound **2**

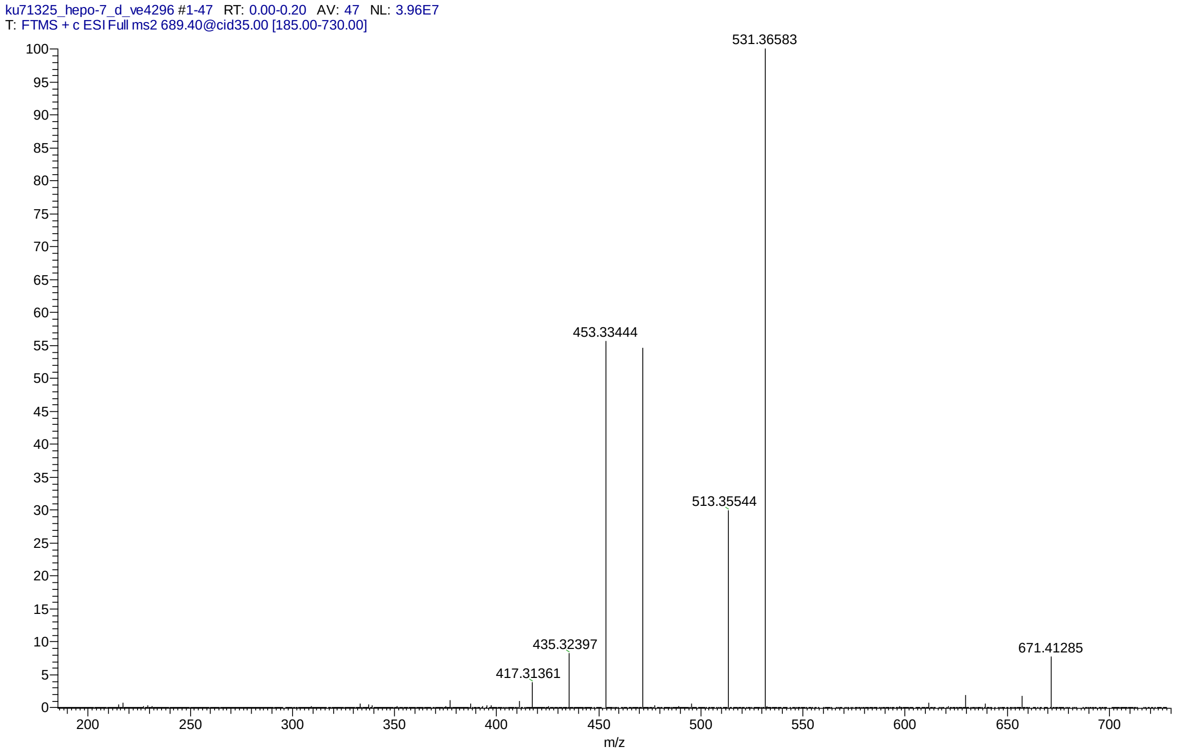

Figure S4. MSMS spectrum of compound **2**

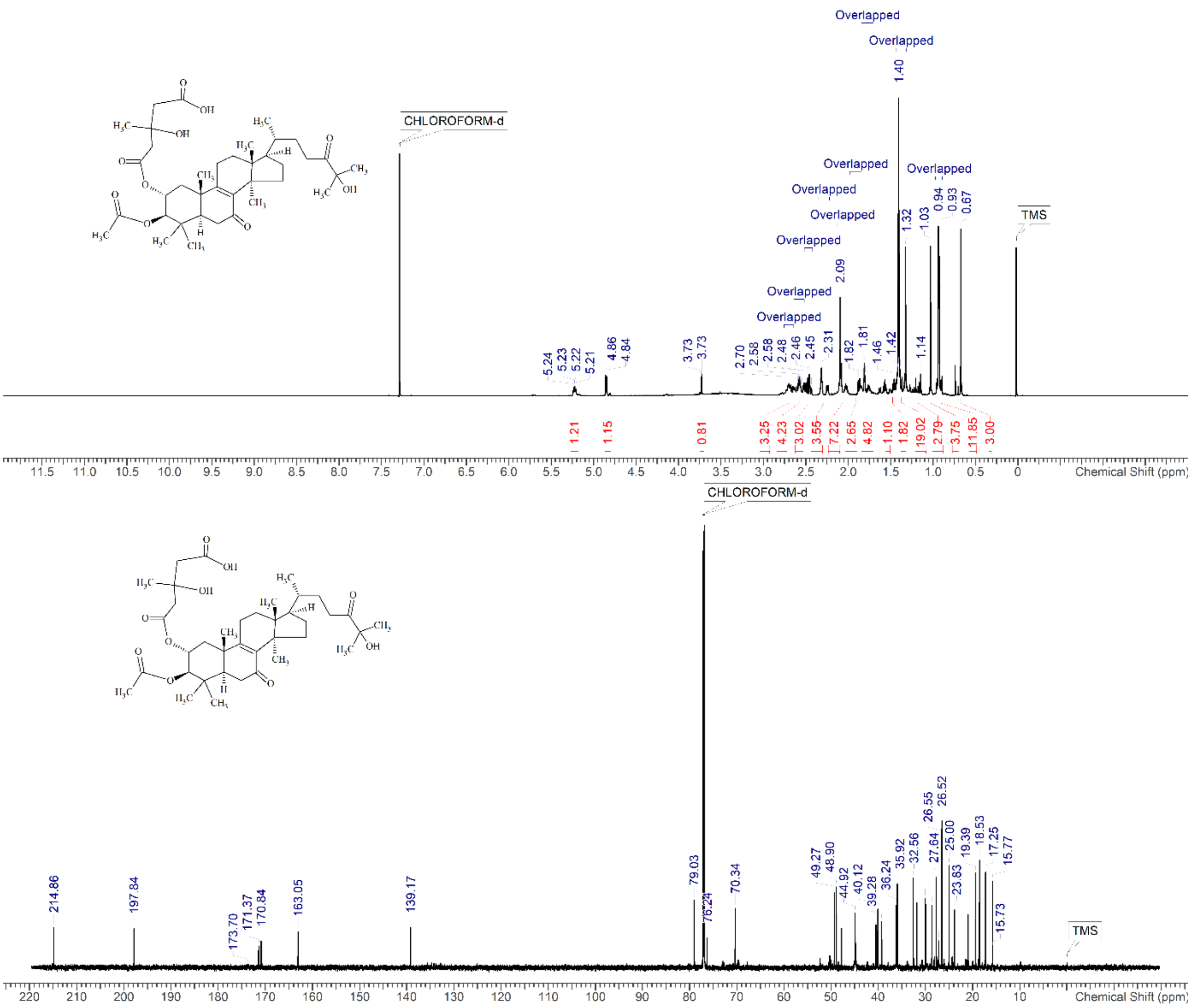

Figure S5. ^{1}H and ^{13}C spectra of compound **1**

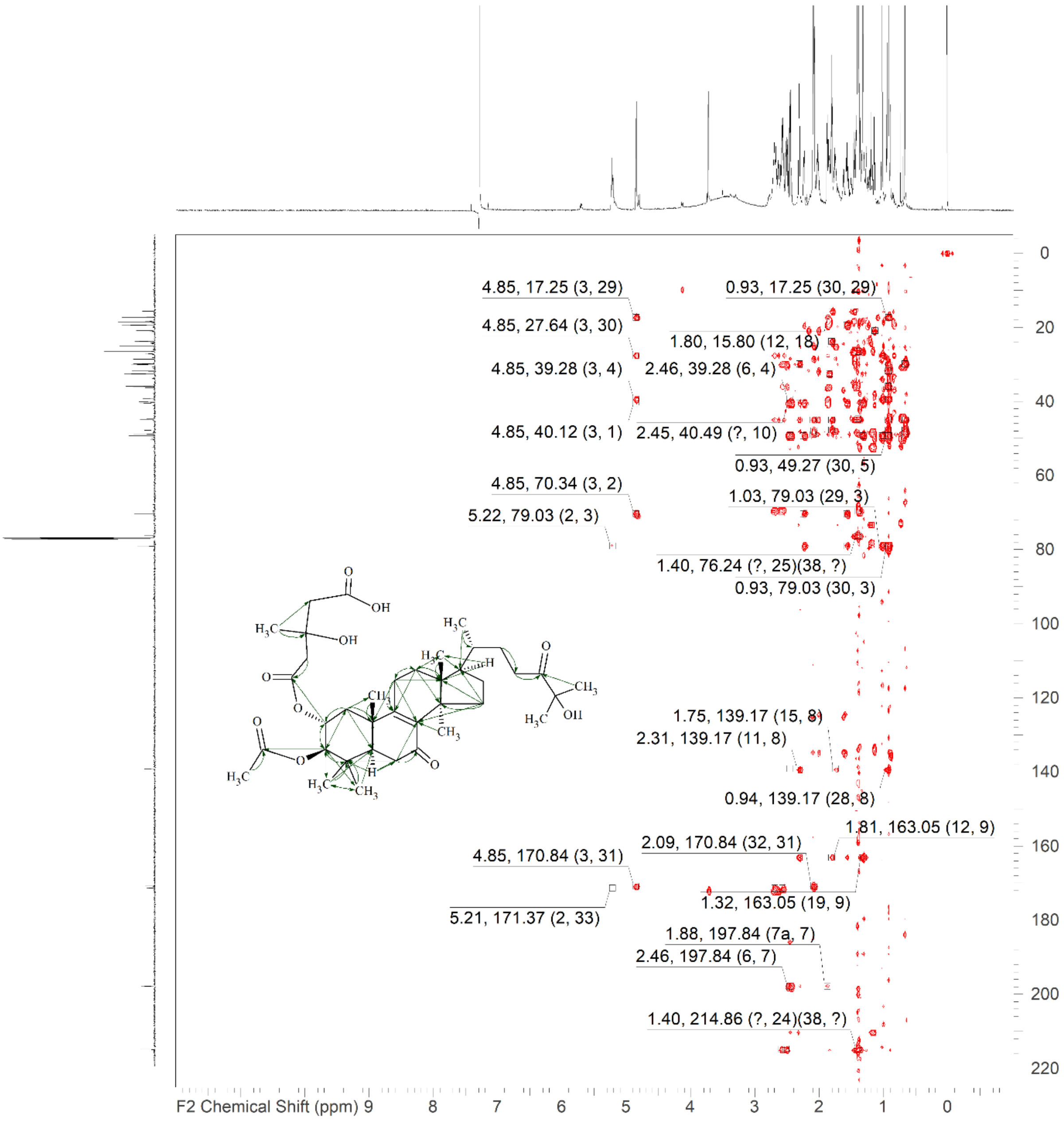

Figure S6. HMBC spectrum of compound **1**

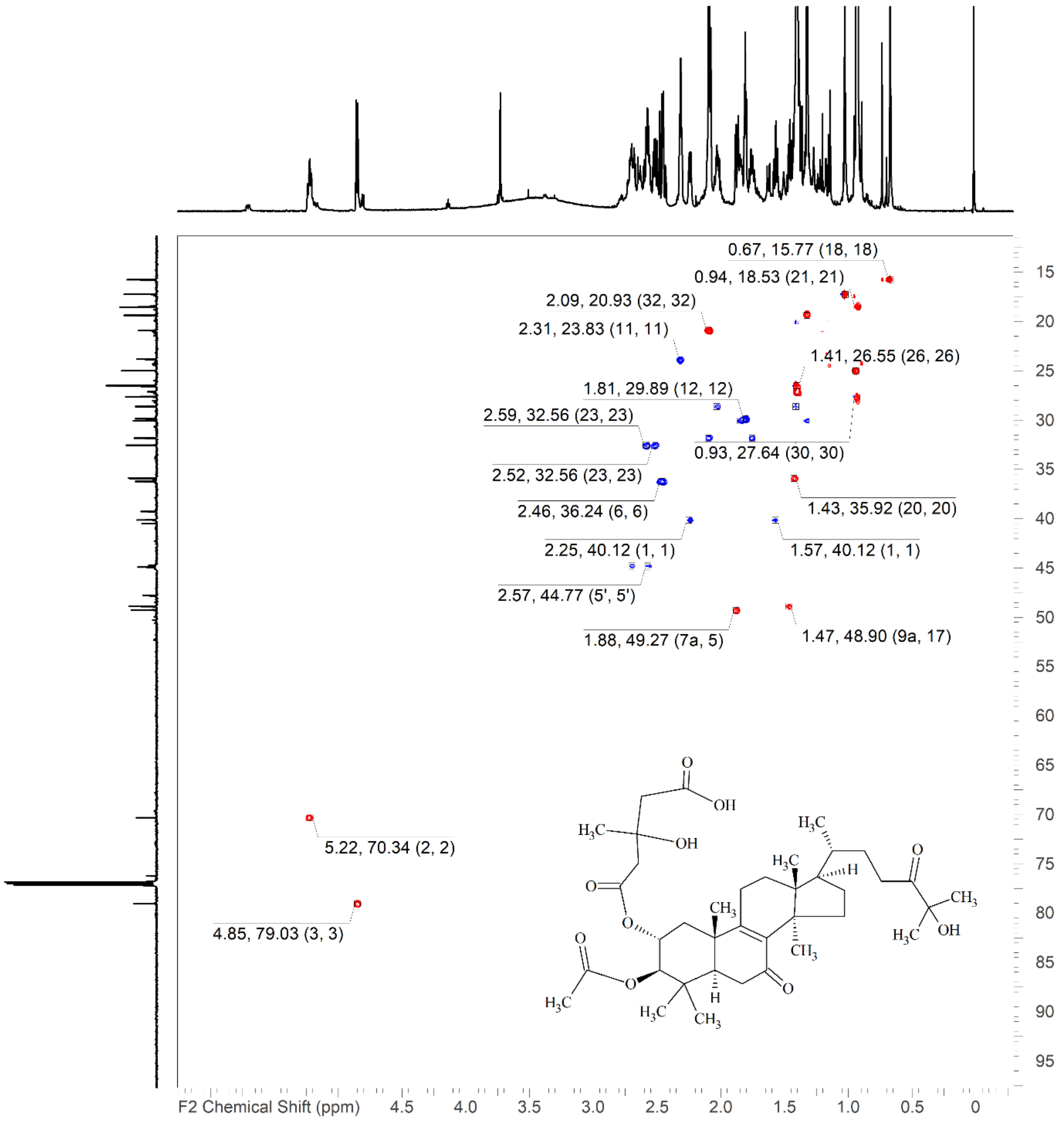

Figure S7. HSQC spectrum of compound **1**

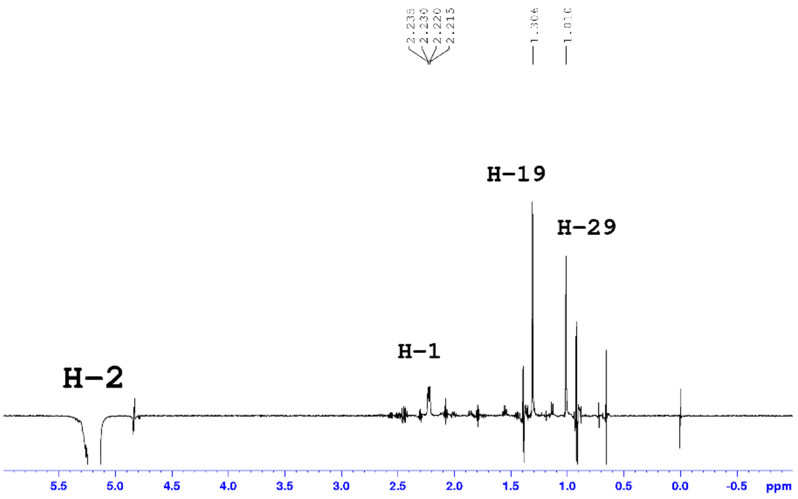

Figure S8. 1D ROESY spectrum of compound **1**

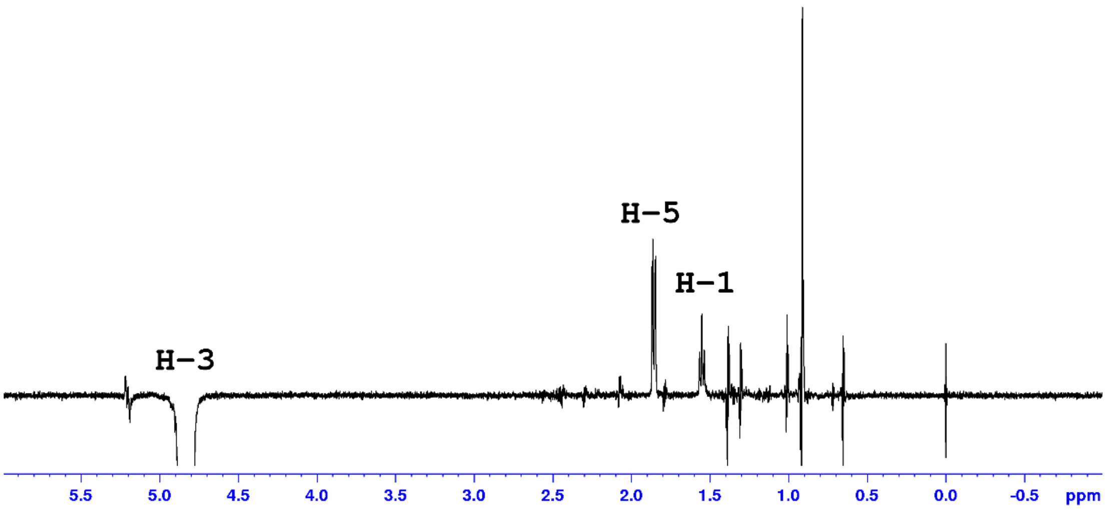

Figure S9. 1D ROESY spectrum of compound **1**

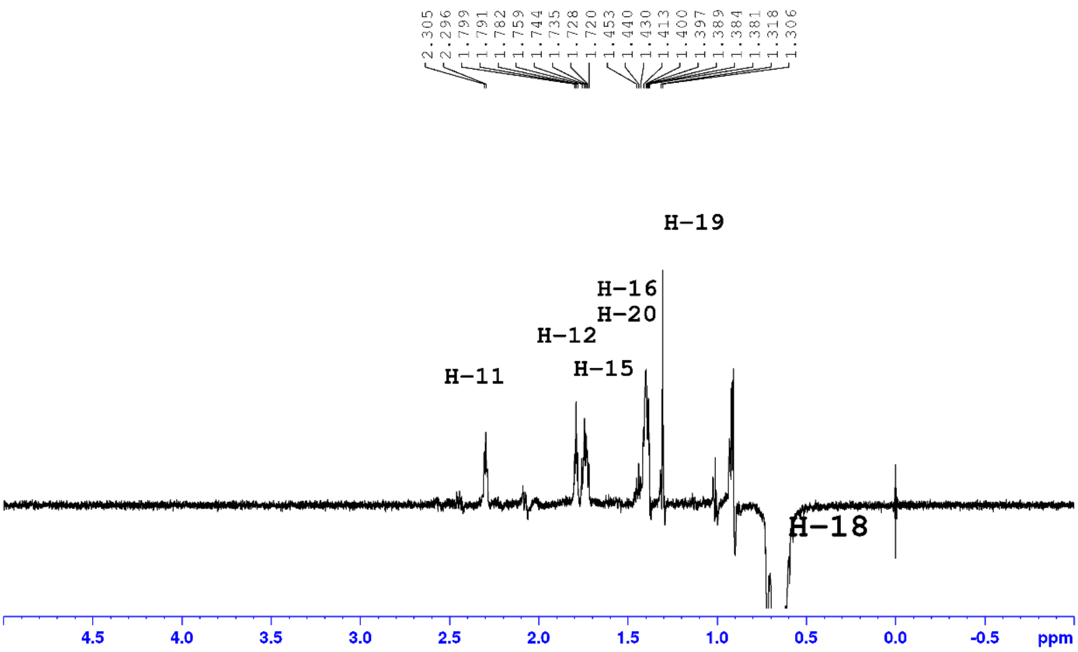

Figure S10. 1D ROESY spectrum of compound **1**

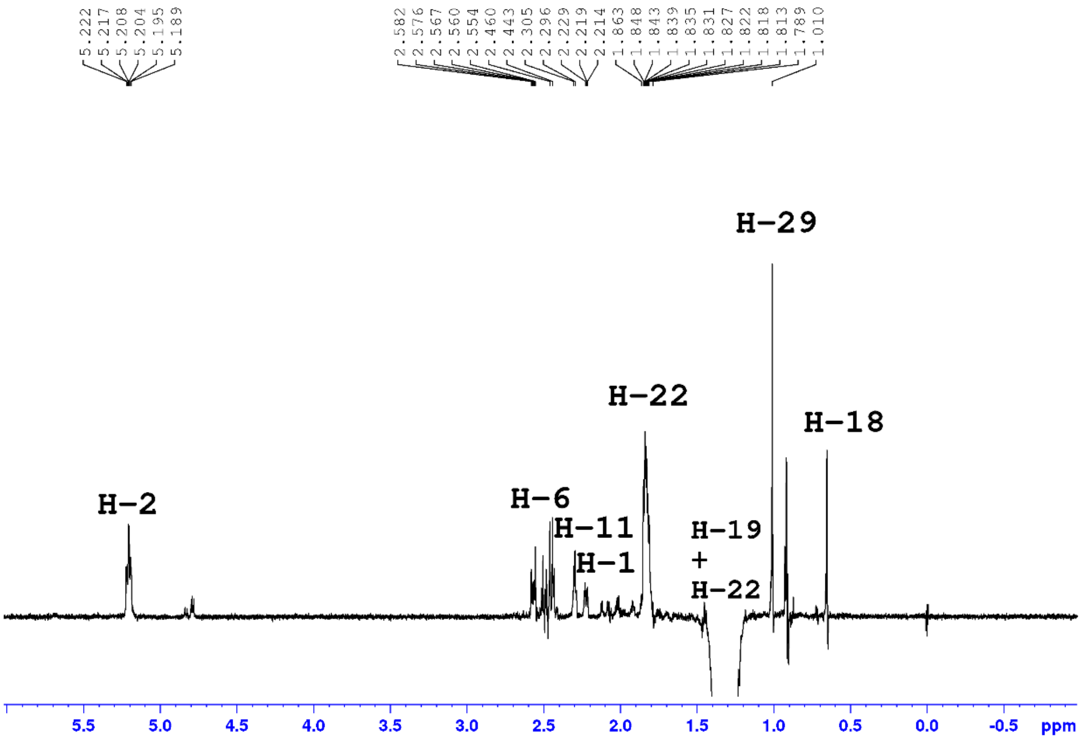

Figure S11. 1D ROESY spectrum of compound **1**

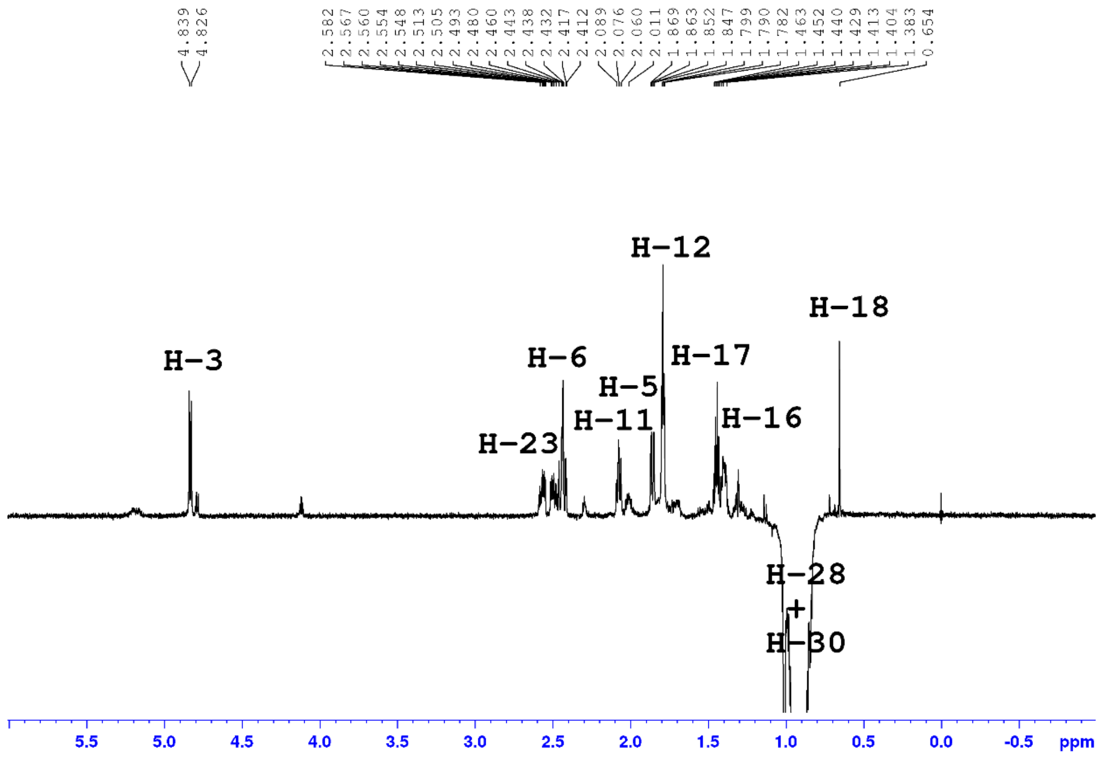

Figure S12. 1D ROESY spectrum of compound **1**

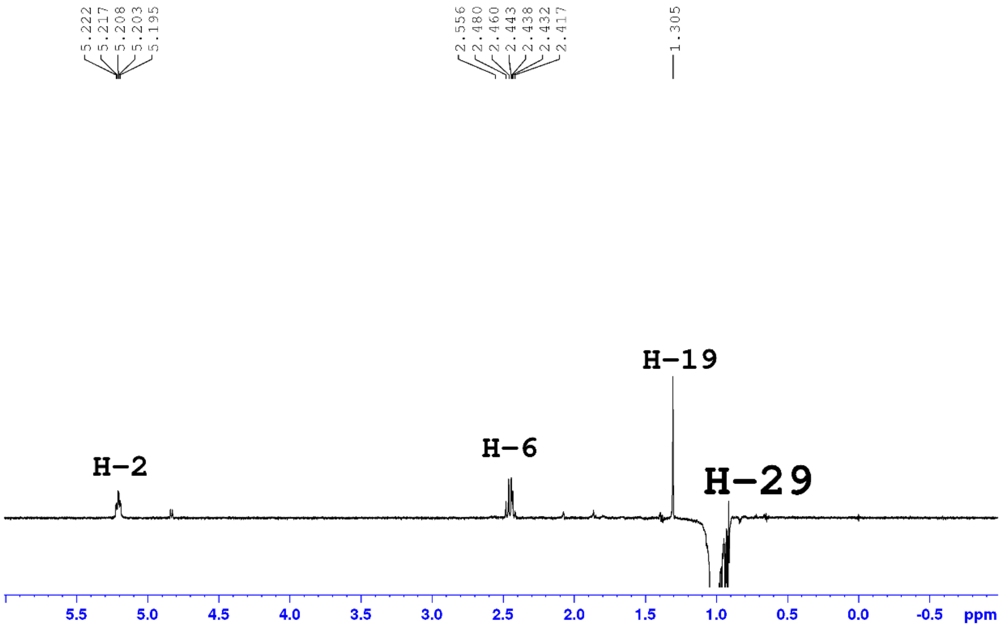

Figure S12. 1D ROESY spectrum of compound **1**

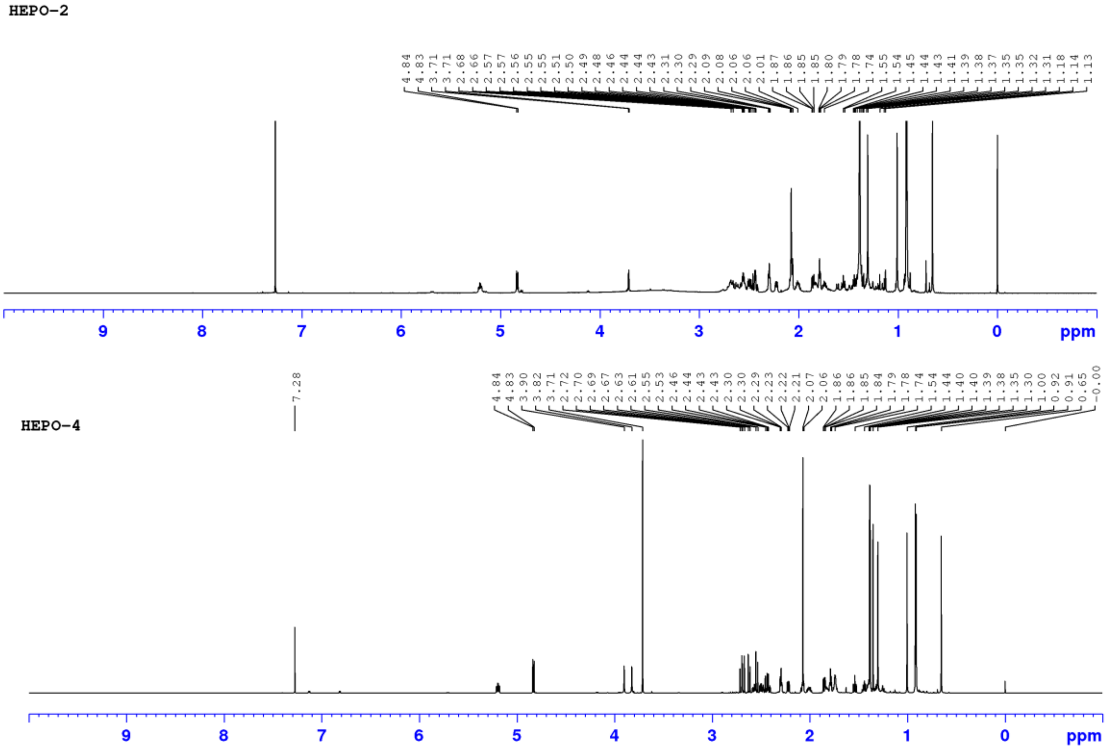

Figure S13. ^{1}H NMR spectra of compounds **1** and **4**

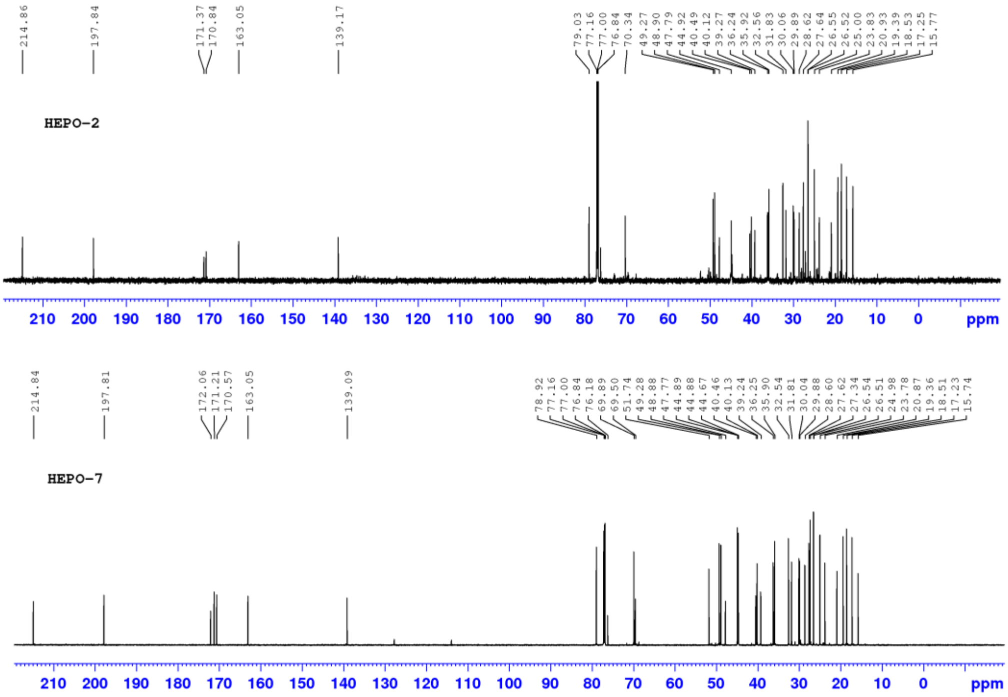

Figure S14. ^{13}C NMR spectra of compounds **1** and **2**

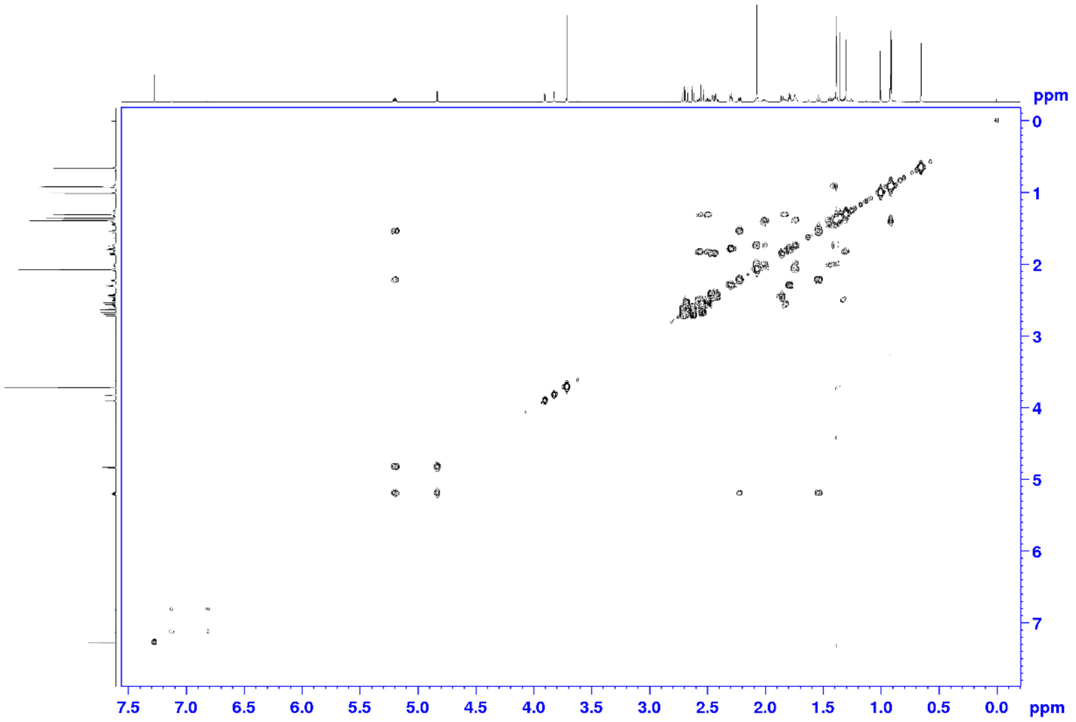

Figure S15. COSY spectrum of compound **2**

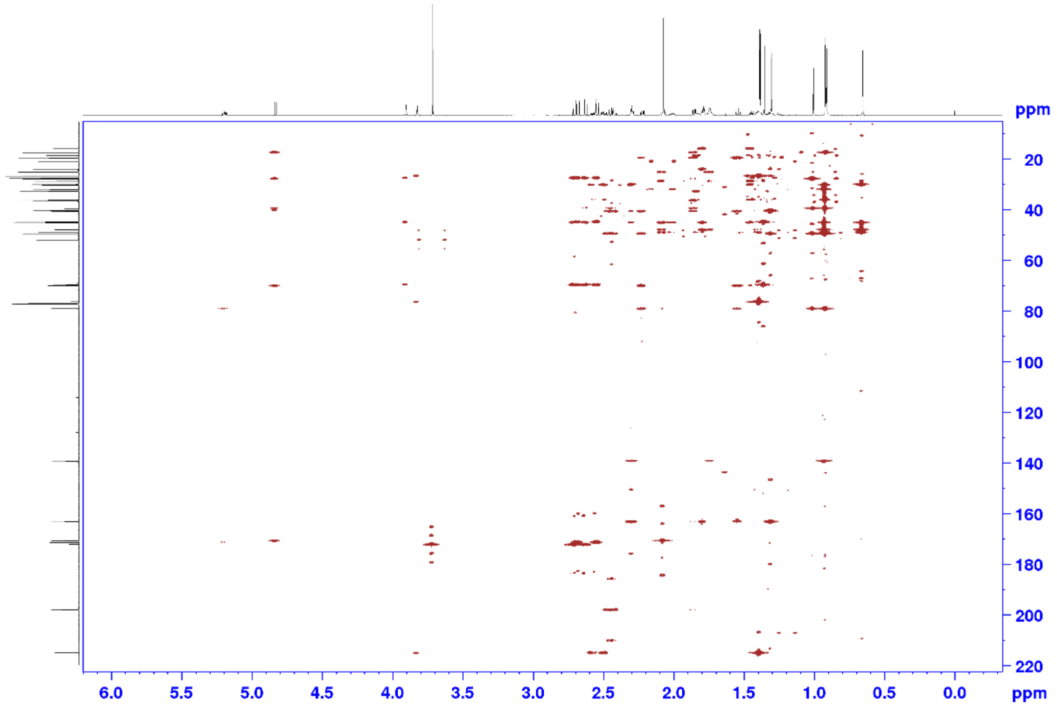

Figure S16. HMBC spectrum of compound **2**

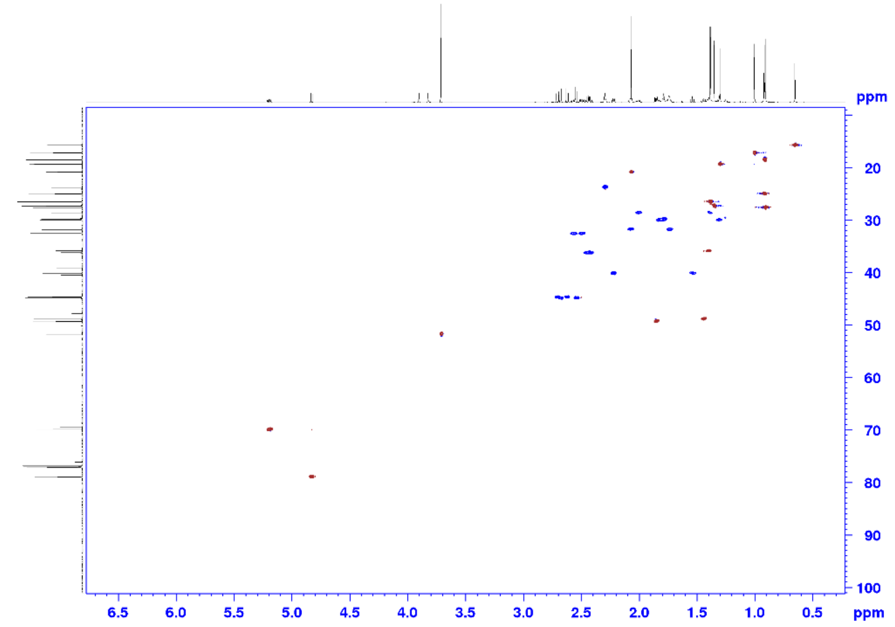

Figure S17. HSQC spectrum of compound **2**

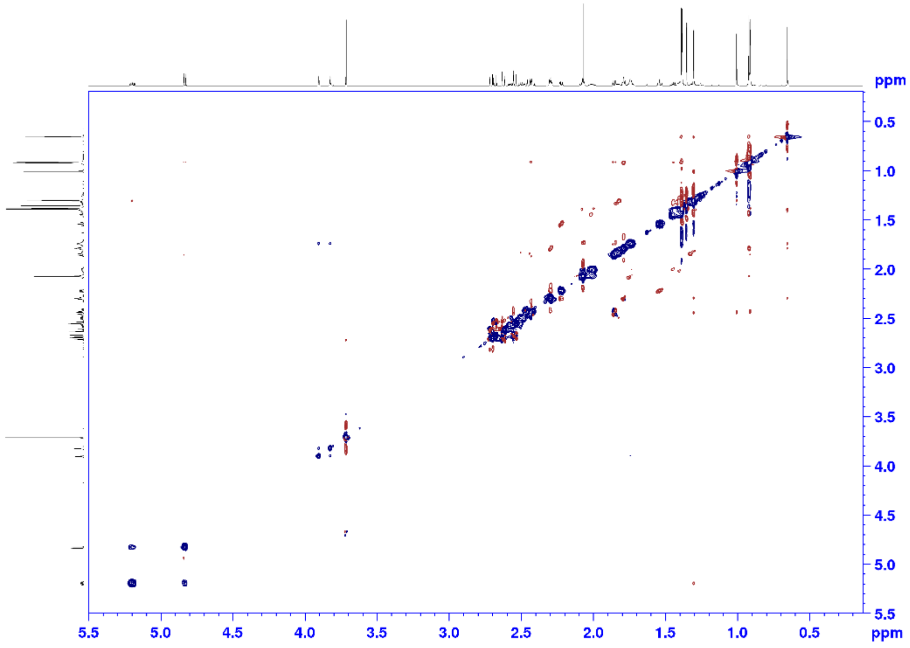

Figure S18. ROESY spectrum of compound **2**

Spectra and spectral data on compounds 3 and 4

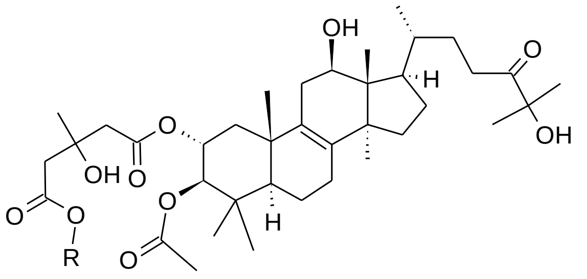

R=H
R=CH_3

Compound **3**

HRMS: M-H=675.41283 (delta=2.2 ppm; $C_{38}H_{59}O_{10}$). HR-ESI-MS-MS (CID=35%; rel. int. %): 613(100); 573(92); 555(6); 531(31).

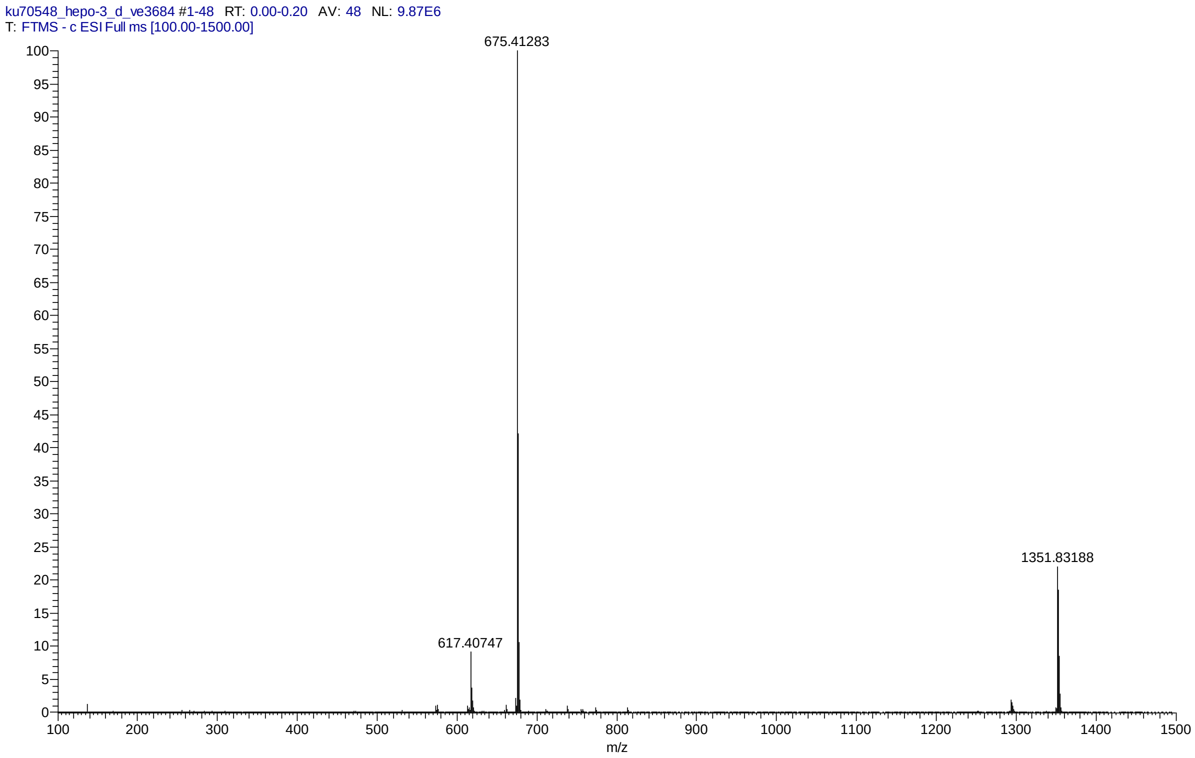

Figure S19. HRMS spectra of compound **3**

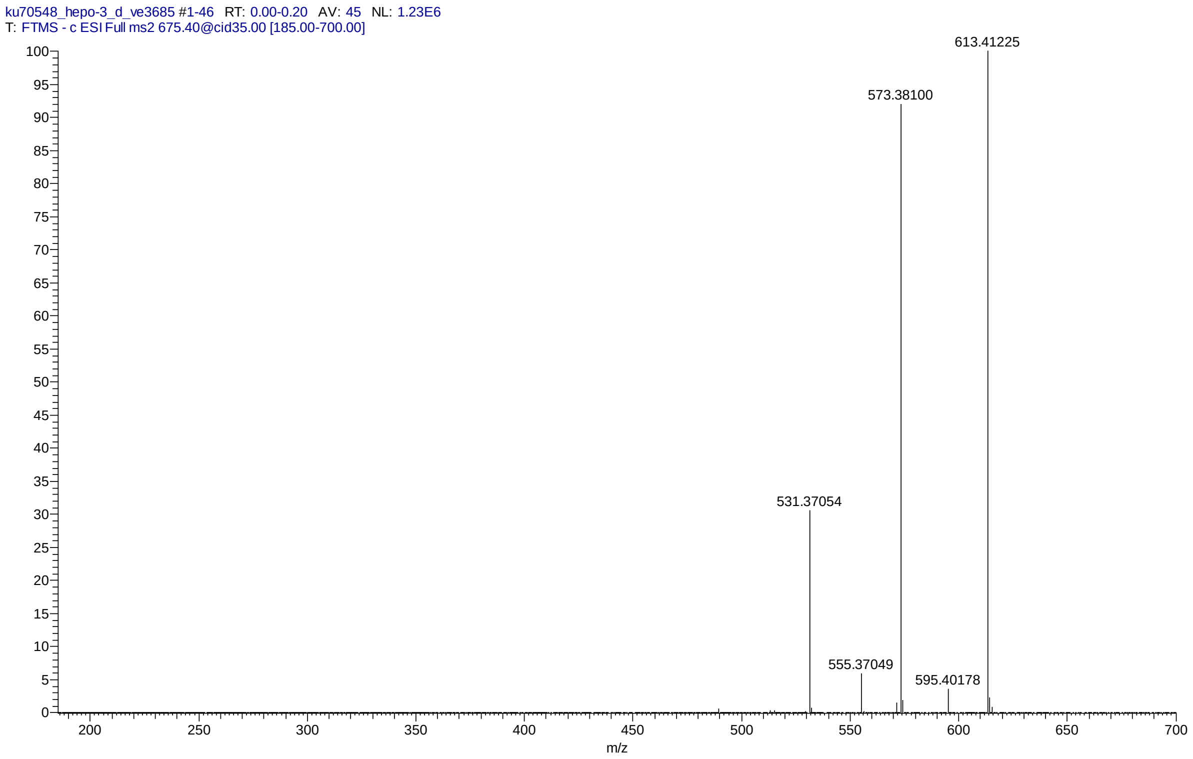

Figure S20. HRMS-MS spectra of compound **3**

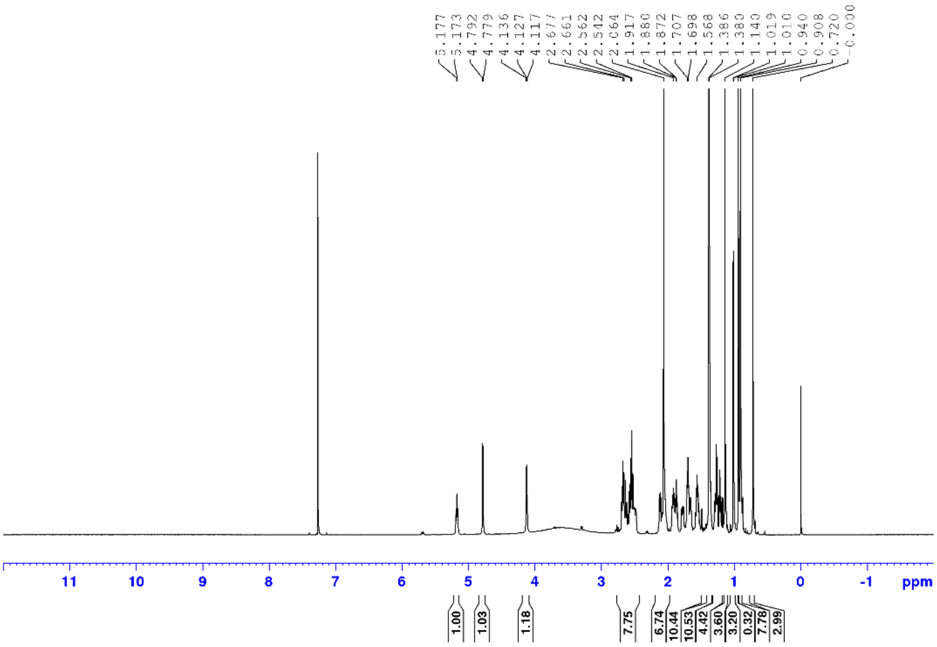

Figure S21. 800 MHz ^{1}H NMR spectrum of compound **3**

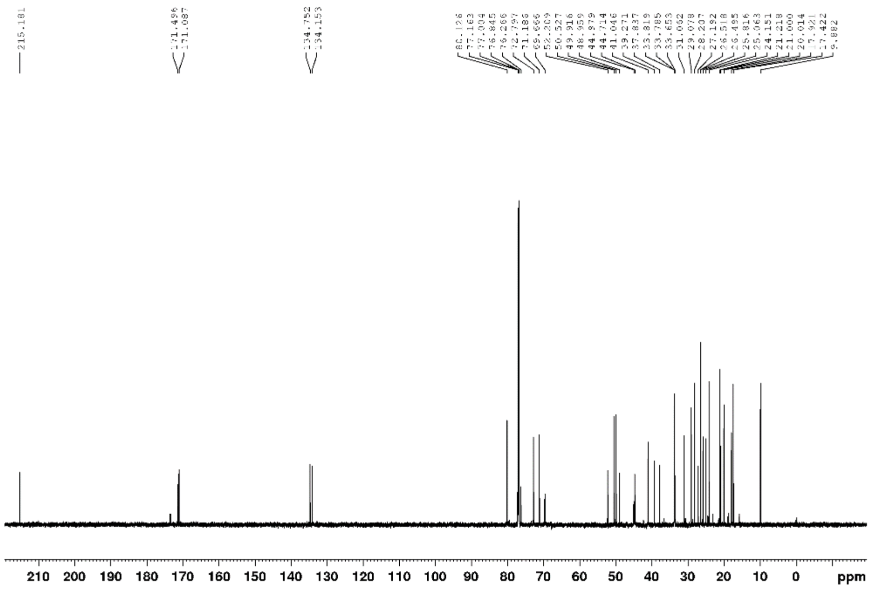

Figure S22. 200 MHz ^{13}C NMR spectrum of compound **3**

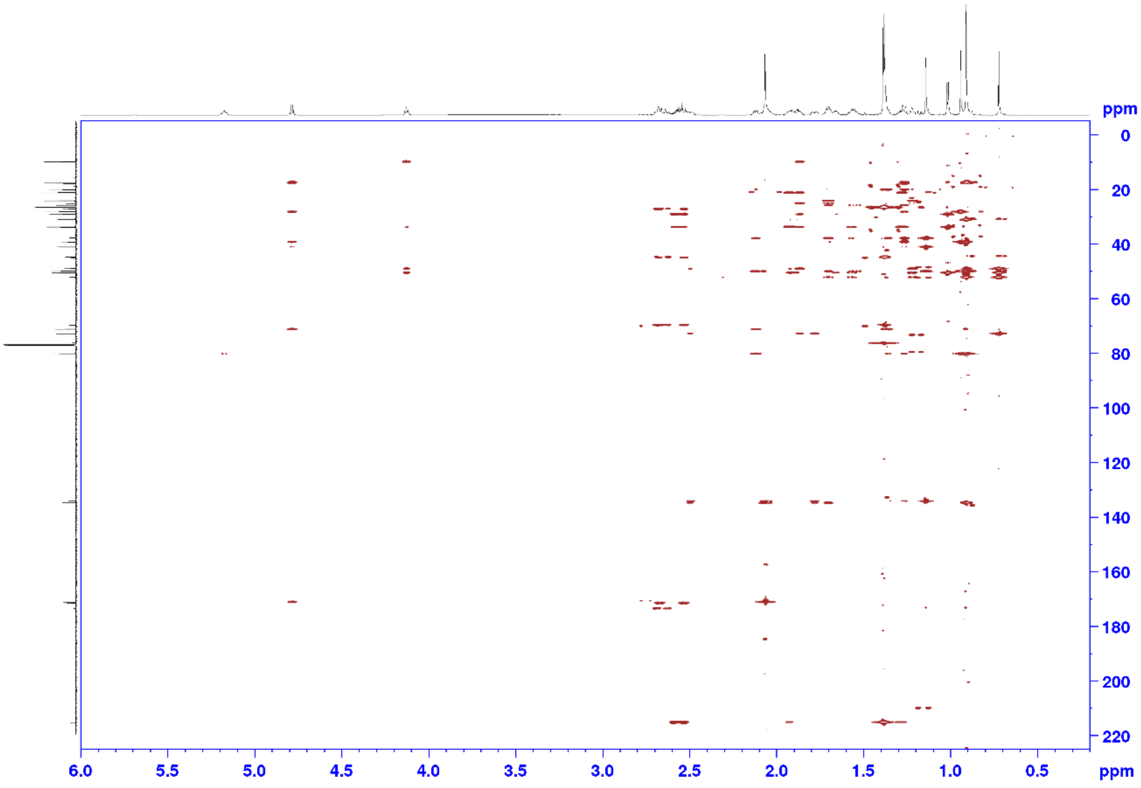

Figure S23. 800 MHz HMBC spectrum of compound **3**

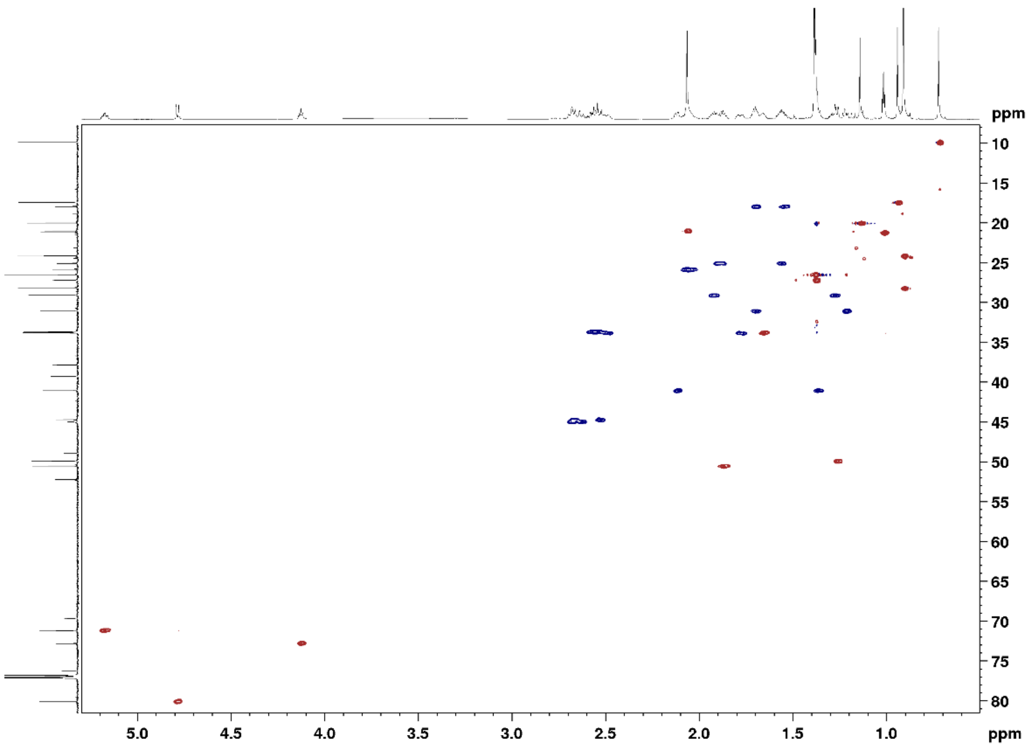

Figure S24. 800 MHz HSQC NMR spectrum of compound **3**

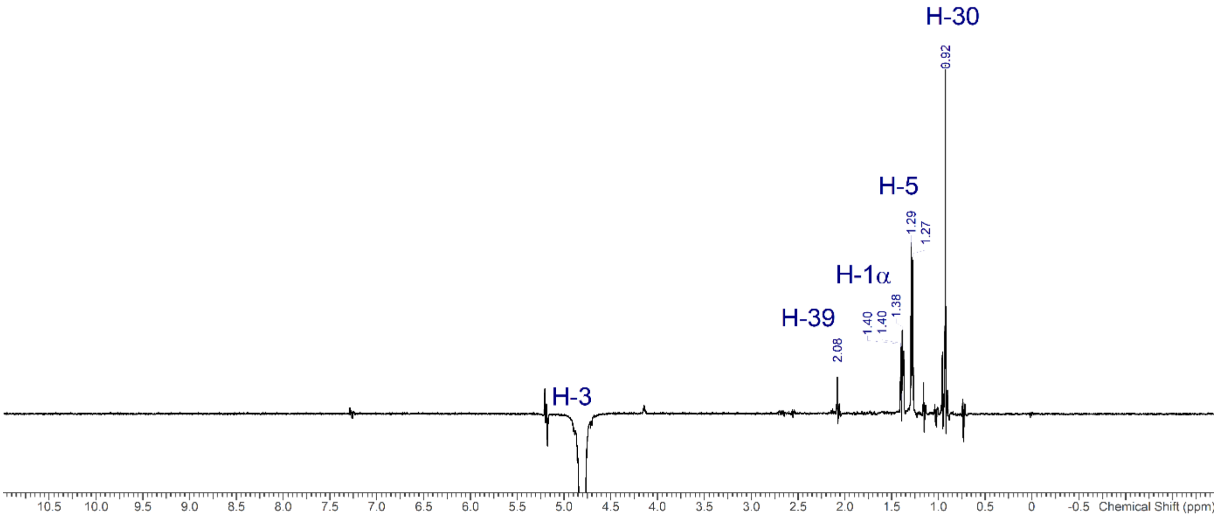

Figure S25. 1D ROESY spectrum of compound **3**

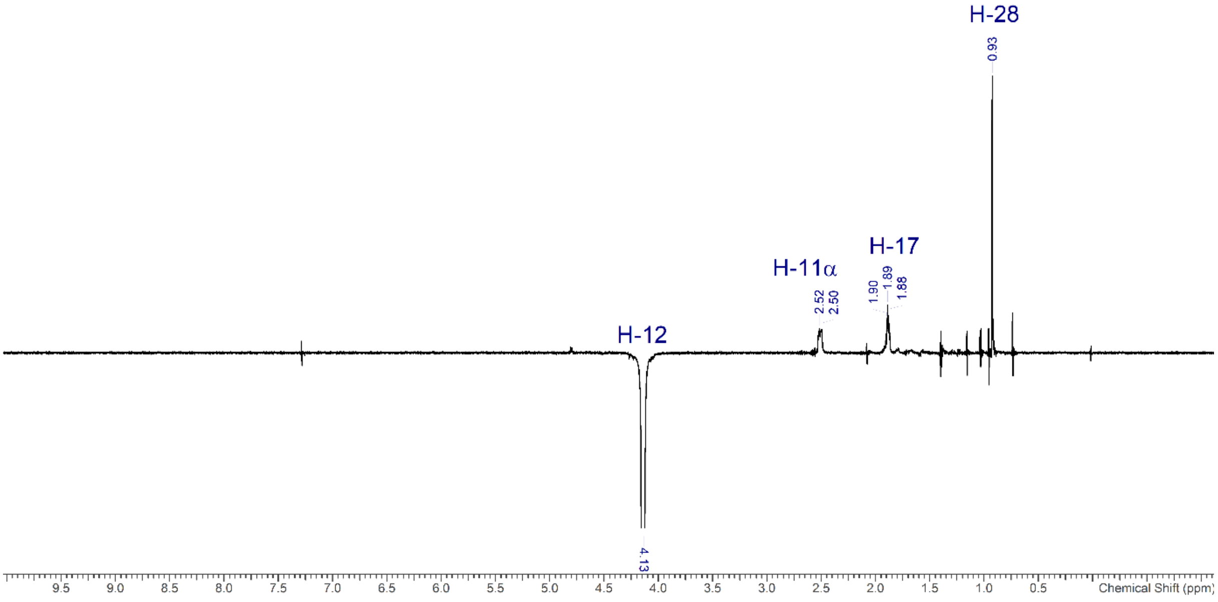

Figure S26. 1D ROESY spectrum of compound **3**

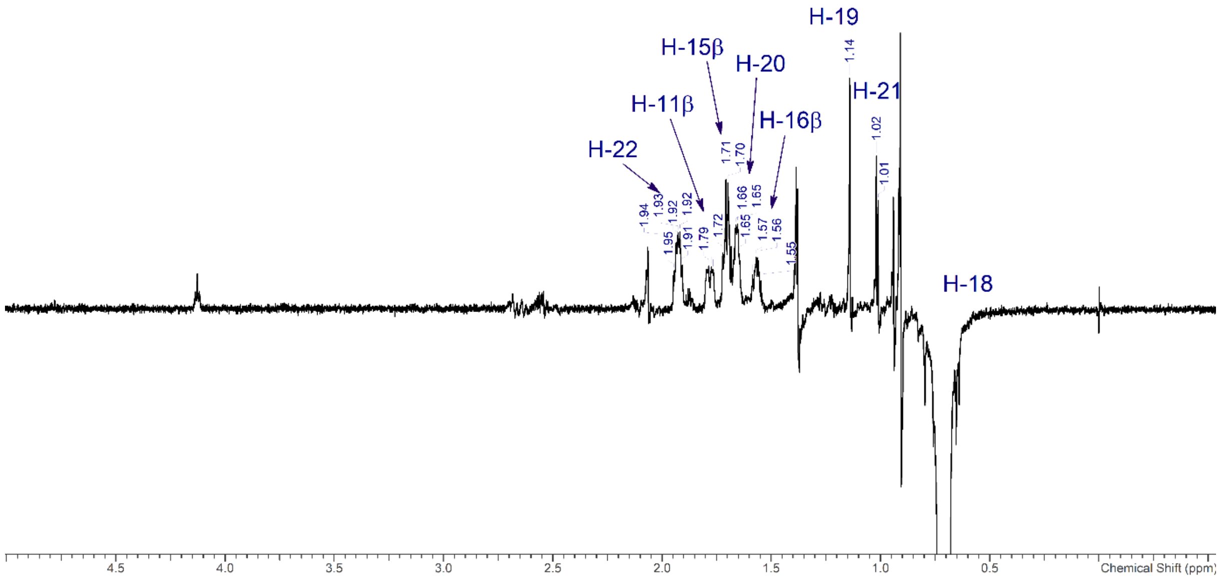

Figure S27. 1D ROESY spectrum of compound **3**

Compound **4**

HRMS: 2M+Na=1403.85130 (delta=-4.6 ppm; $C_{78}H_{124}O_{20}Na$). HR-ESI-MS^3 (1408/713; CID=35%, 45%; rel. int. %): 653(12); 639(6); 537(100); 477(41); 437(3).

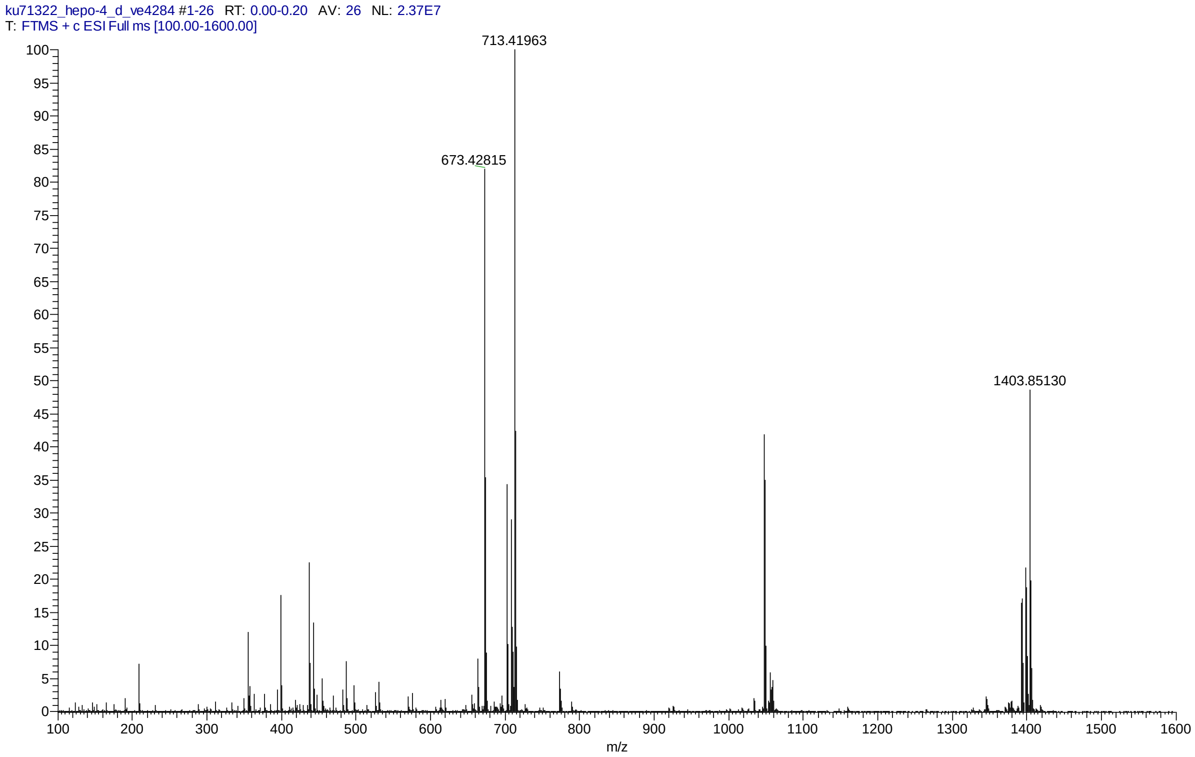

Figure S28. HRMS spectra of compound **4**

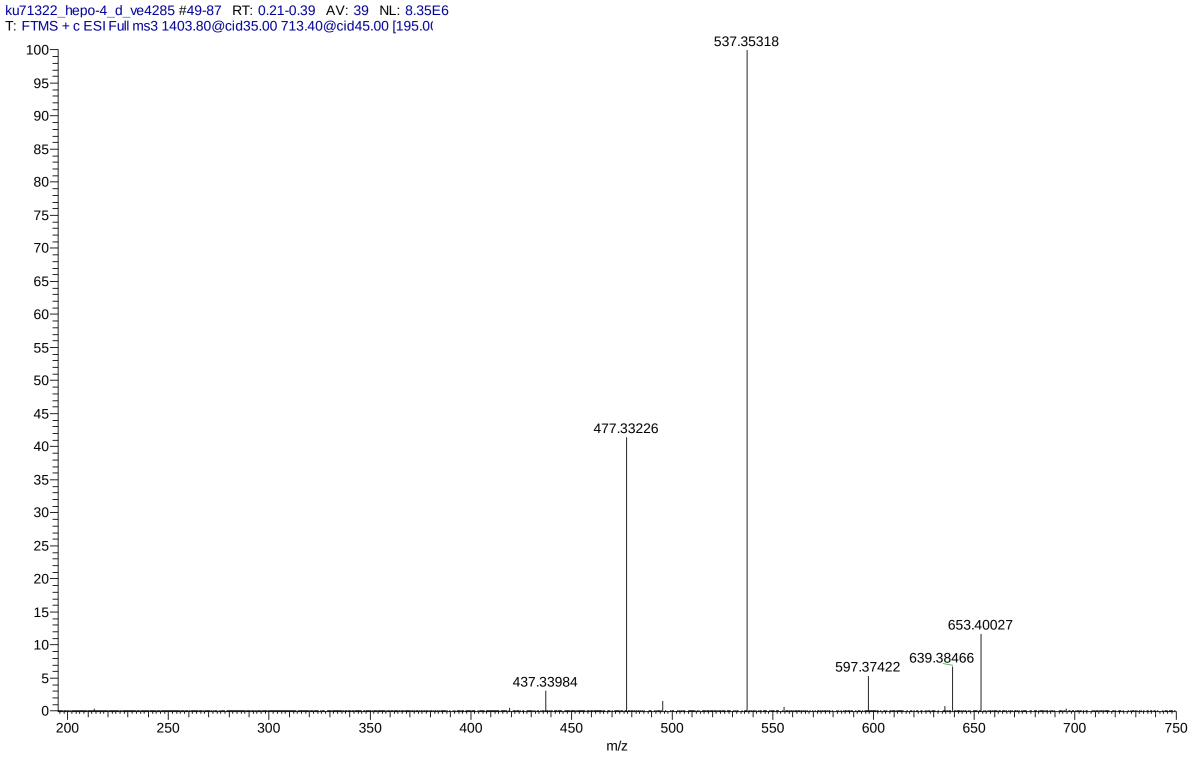

Figure S29. HRMS-MS spectra of compound **4**

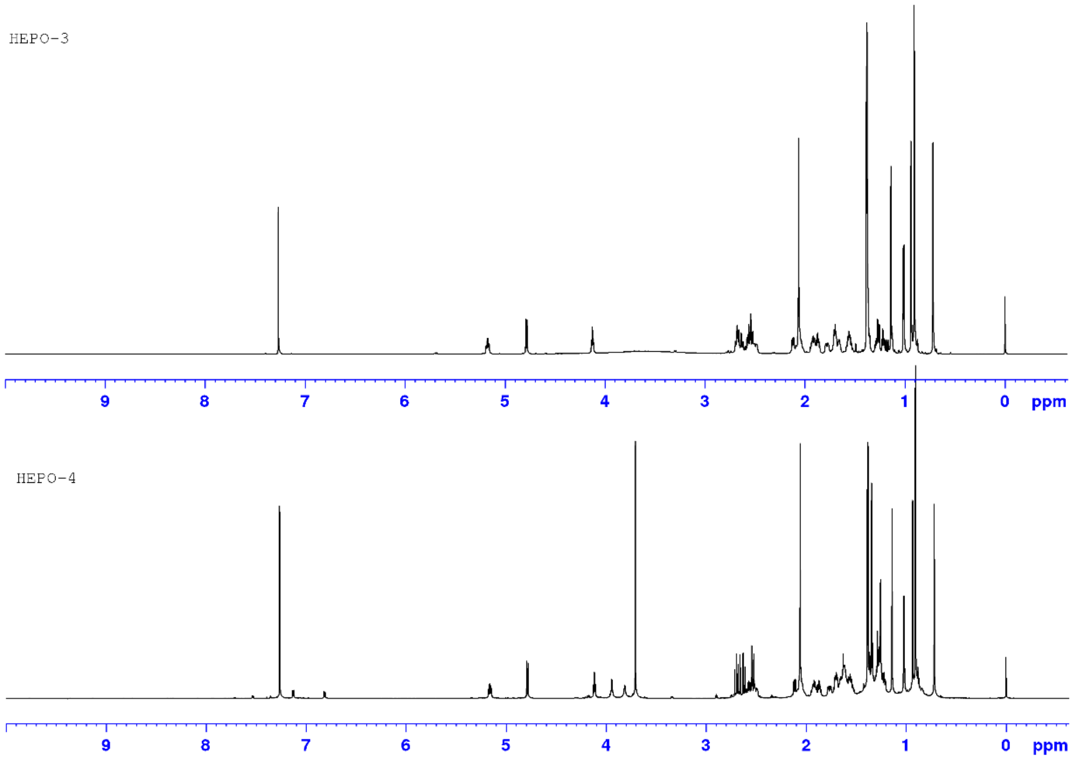

Figure S30. 800 MHz ^{1}H NMR spectra of compounds **3** (top) and **4** (bottom)

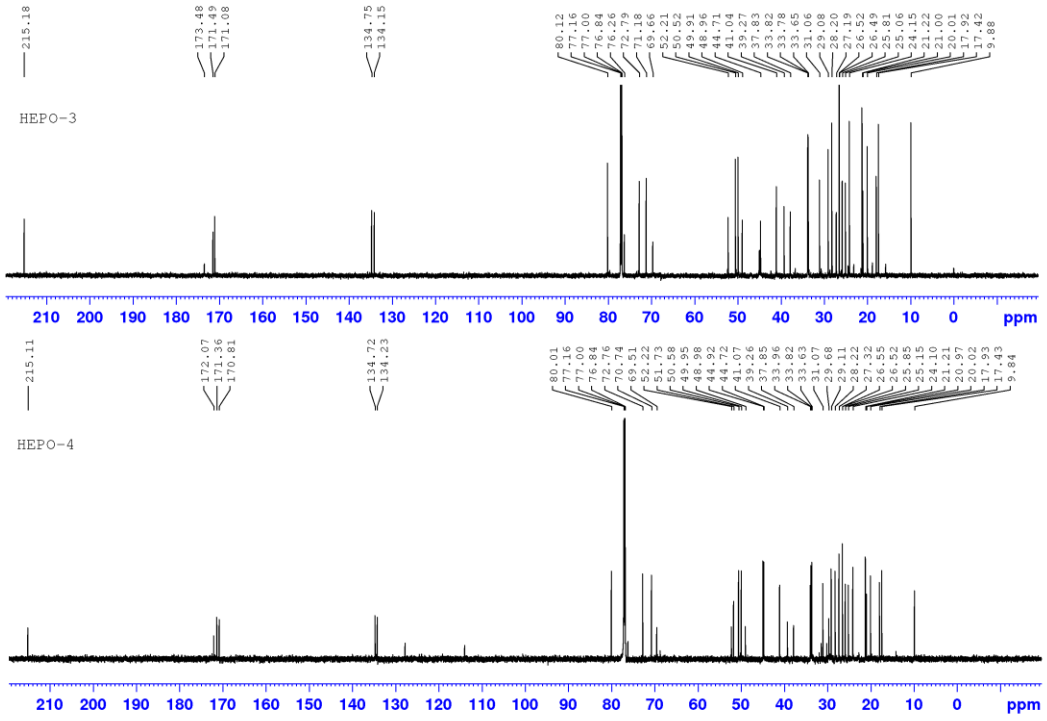

Figure S31. 200 MHz ^{13}C NMR spectra of compounds **3** (top) and **4** (bottom)

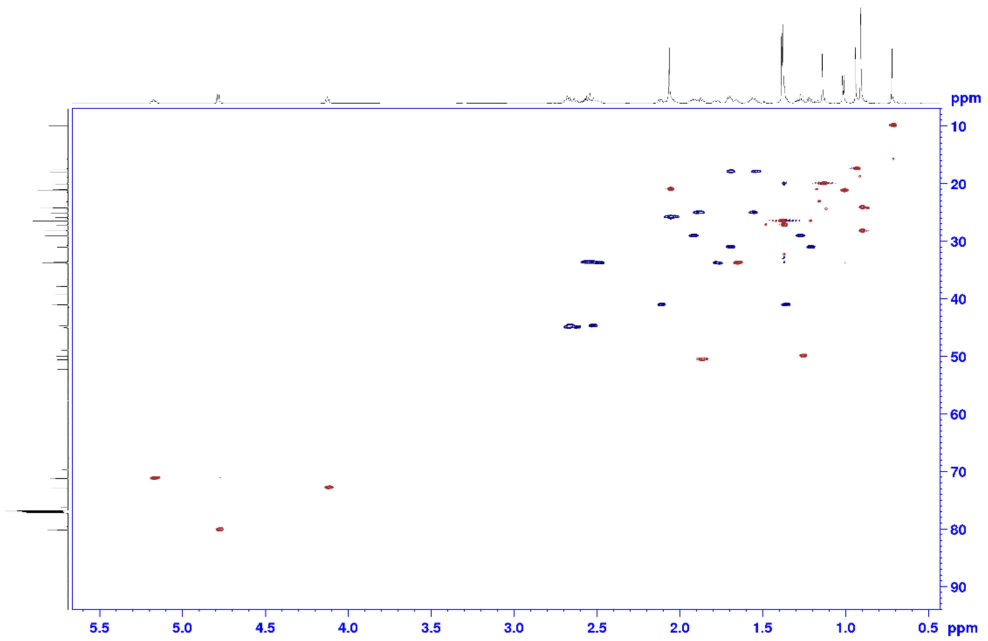

Figure S32. HSQC spectrum of compound **4**

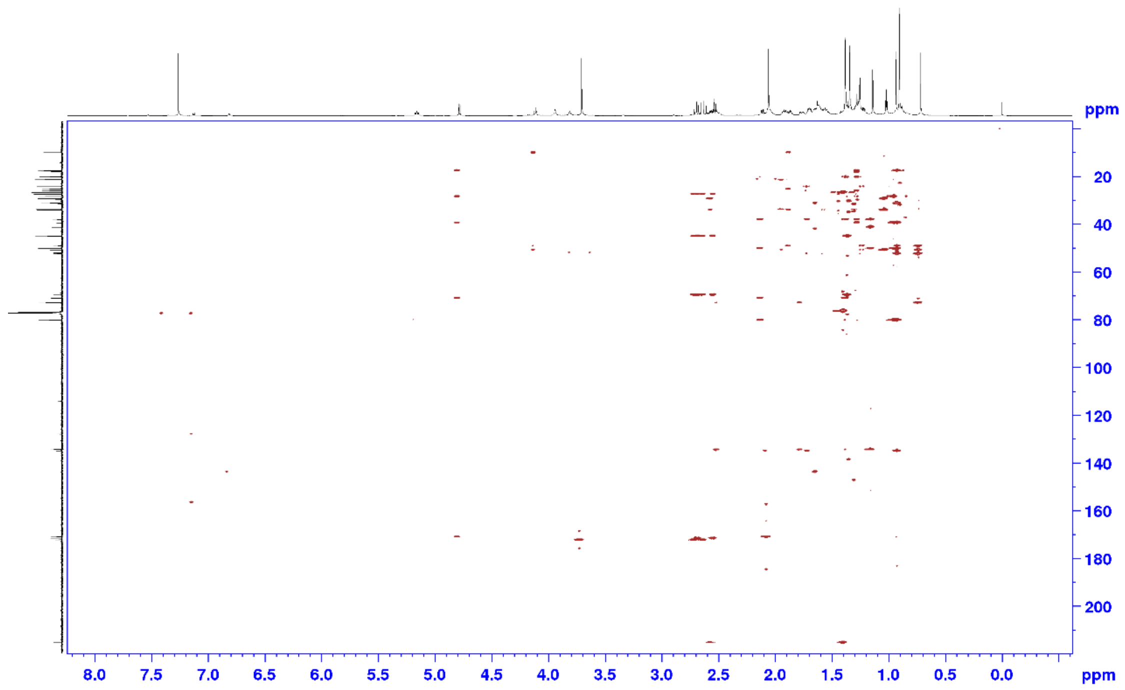

Figure S33. HMBC spectrum of compound **4**

Compound **5**

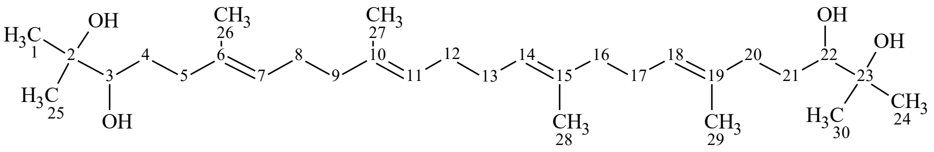

HRMS: M+H=479.40900 (delta=-1.0 ppm; $C_{30}H_{55}O_5$). HR-ESI-MS-MS (CID=35%; rel. int. %): 461(100); 443(31).

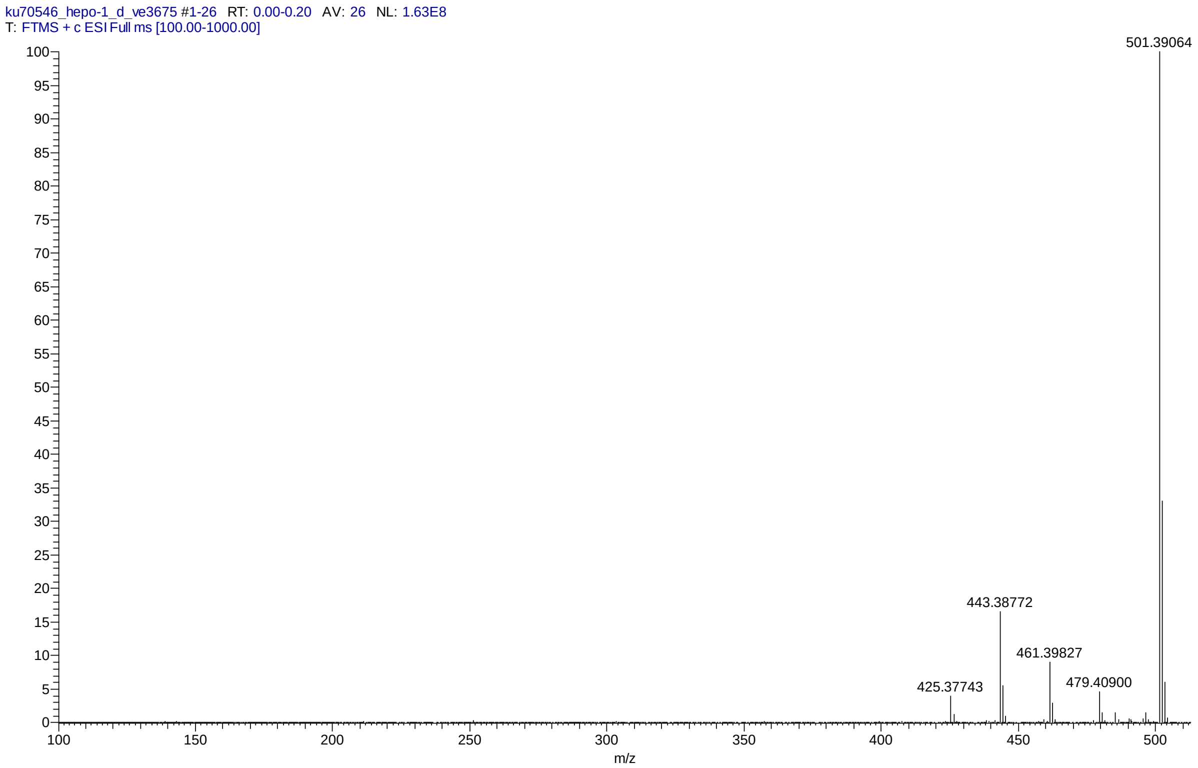

Figure S34. ESI-HR-MS spectrum of compound **5**

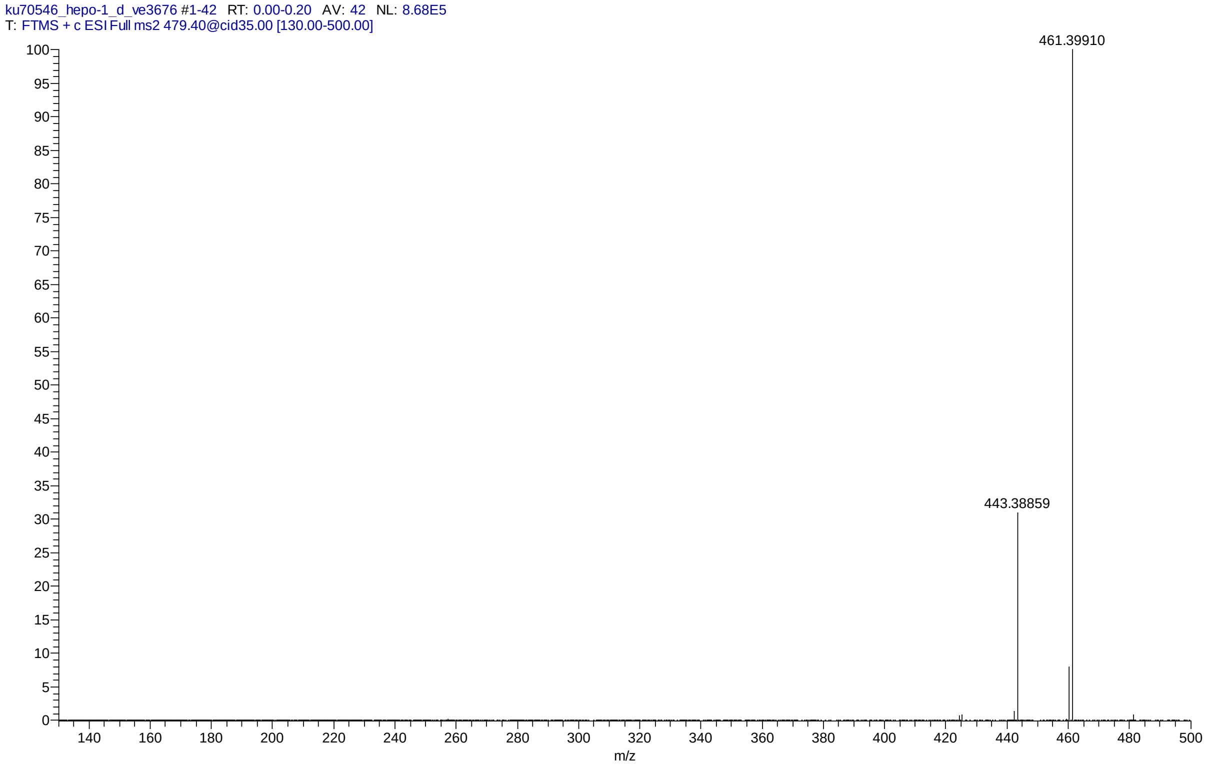

Figure S35. ESI-HR-MS-MS of compound **5**

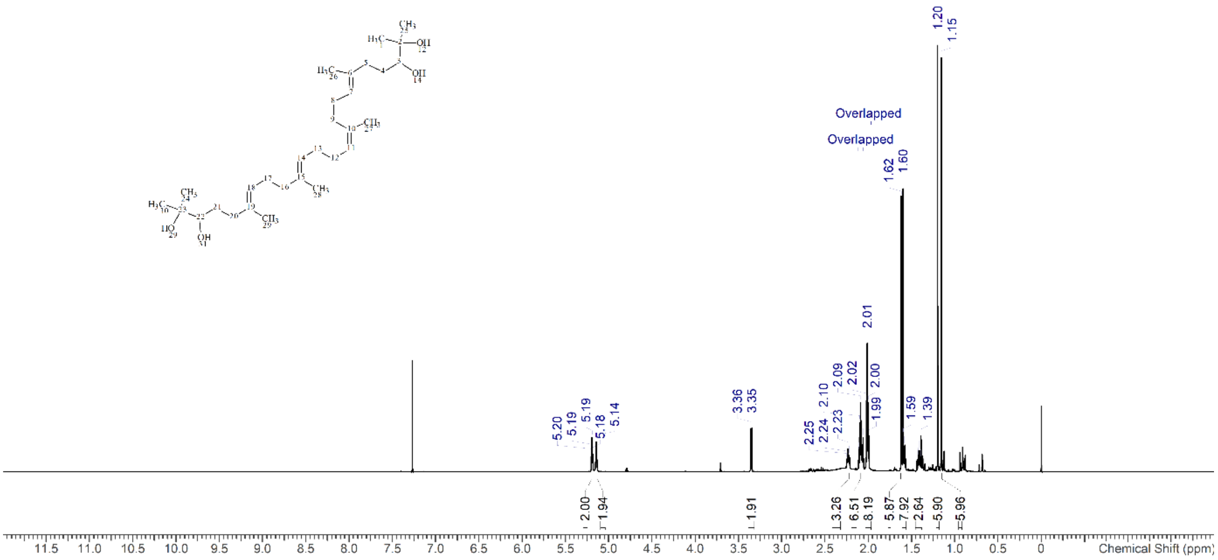

Figure S36. ^{1}H NMR spectrum of compound **5**

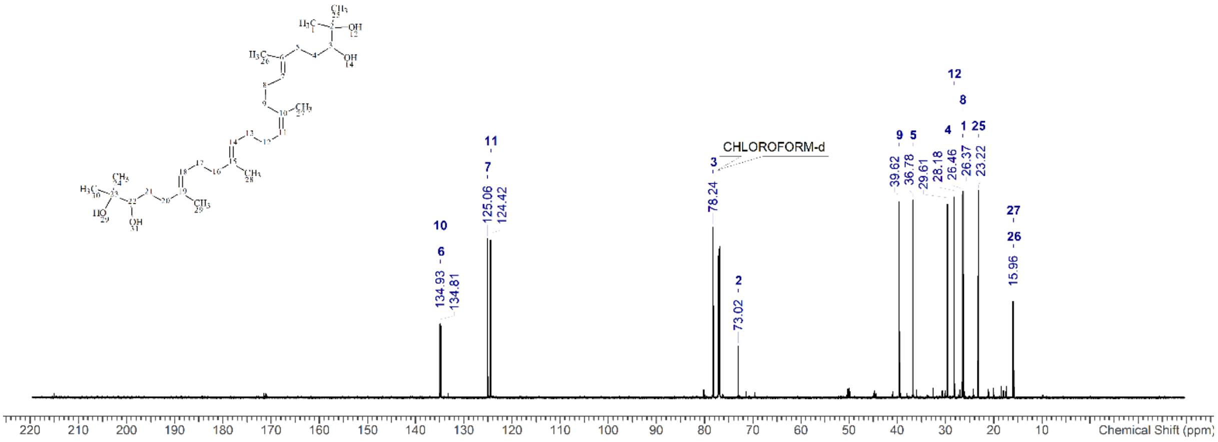

Figure S37. ^{13}C NMR spectrum of compound **5**

Table S1. ^{1}H and ^{13}C NMR assignments of **5** at 800 MHz in $CDCl_3$

Pos.	^{13}C	^{1}H	Mult. (J in Hz)
1, 24 & 25,30	23.2 & 26.4	1.15 & 1.20	s
2	73.0	-	
3	78.2	3.35	dd (10.5, 2.0)
4	29.6	1.41	m
4	29.6	1.58	m
5	36.8	2.23	m
5	36.8	2.07	m
6	134.8	-	
7	125.1	5.19	ddq (8.2, 5.8, ~1.1)
8	26.5	2.09	m
9	39.6	2.00	m
10	134.9	-	
11	124.4	5.14	ddq (5.3, 2.5, ~1.1)
12	28.2	2.01	m
26, 29	15.9	1.62	d (~1.0)
27, 28	16.0	1.60	d (1.1)

Table S2. Cytotoxic Effect of the Compounds (**1-6**) Isolated from *H. populnea*

**Compounds**	**Colo205 (IC_{50} µM)**		**Colo320 (IC_{50} µM)**		**MRC-5 (IC_{50} µM)**	
	**Mean**	**SD**	**Mean**	**SD**	**Mean**	**SD**
Pholiol A (**1**)	>100	-	>100	-	>100	-
Pholiol B (**2**)	67.92	0.39	61.5	4.7	89.96	0.01
Pholiol C (**3**)	>100	-	>100	-	>100	-
Pholiol D (**4**)	51.36	0.1	48.94	0.65	54.18	3.002
Compound **5**	26.7	0.33	27.48	1.56	29.06	2.2
Ergosterol (**6**)	**4.88**	0.57	**6.48**	0.22	**0.50**	0.09
**Doxorubicin**	2.46	0.26	7.44	0.2	>20	-

Table S3. P-gp Efflux Pump Inhibitory Activity of Compounds **1-3**, **5** and **6** against MDR COLO 320 Colon Adenocarcinoma Cells

**Samples**	**conc. µM**	**FSC**	**SSC**	**FL-1**	**FAR**	**Peak Ch**
Tariquidar*	0.2	1945	837	64.100	5.533	58.300
Pholiol A (**1**)	20	2056	833	39.600	3.418	42.200
Pholiol B (**2**)	20	2058	860	79.700	**6.880**	77.700
Pholiol C (**3**)	20	2055	842	11.200	0.967	18.400
Compound **5**	20	2011	887	76.900	**6.638**	93.100
Ergosterol (**6**)	2	2117	837	12.200	1.053	8.350
DMSO	2.00%	2073	848	9.590	0.828	4.530
Colo 320	-	2052	841	8.870	-	4.530

*Positive control
